# Virtual Reality Smartphone-Based Intervention for Smoking Cessation: Pilot Randomized Controlled Trial on Initial Clinical Efficacy and Adherence

**DOI:** 10.2196/17571

**Published:** 2020-07-29

**Authors:** Emilio Goldenhersch, Johannes Thrul, Joaquín Ungaretti, Nicolas Rosencovich, Cristian Waitman, Marcelo Rodriguez Ceberio

**Affiliations:** 1 Laboratorio de Investigación en Neurociencia y Ciencias Sociales Universidad de Flores Ciudad Autónoma de Buenos Aires Argentina; 2 Department of Mental Health Johns Hopkins Bloomberg School of Public Health Baltimore, MD United States; 3 Facultad de Psicología Universidad de Buenos Aires Buenos Aires Argentina; 4 Consejo Nacional de Investigaciones Científicas y Técnicas Buenos Aires Argentina; 5 Escuela de Ingeniería Biomédica Universidad Nacional de Córdoba Córdoba Argentina; 6 Universidad Siglo XXI Córdoba Argentina; 7 Departamento de Psicología Universidad de Flores Buenos Aires Argentina; 8 Escuela Sistemica de Psicología Buenos Aires Argentina

**Keywords:** smoking cessation, nicotine dependence, craving, virtual reality, mindfulness, digital therapy, mHealth, mobile phone

## Abstract

**Background:**

Obstacles to current tobacco cessation programs include limited access and adherence to effective interventions. Digital interventions offer a great opportunity to overcome these difficulties, yet virtual reality has not been used as a remote and self-administered tool to help increase adherence and effectiveness of digital interventions for tobacco cessation.

**Objective:**

This study aimed to evaluate participant adherence and smoking cessation outcomes in a pilot randomized controlled trial of the digital intervention Mindcotine (MindCotine Inc) using a self-administered treatment of virtual reality combined with mindfulness.

**Methods:**

A sample of 120 participants was recruited in the city of Buenos Aires, Argentina (mean age 43.20 years, SD 9.50; 57/120, 47.5% female). Participants were randomly assigned to a treatment group (TG), which received a self-assisted 21-day program based on virtual reality mindful exposure therapy (VR-MET) sessions, daily surveys, and online peer-to-peer support moderated by psychologists, or a control group (CG), which received the online version of the smoking cessation manual from the Argentine Ministry of Health. Follow-up assessments were conducted by online surveys at postintervention and 90-day follow-up. The primary outcome was self-reported abstinence at postintervention, with missing data assumed as still smoking. Secondary outcomes included sustained abstinence at 90-day follow-up, adherence to the program, and readiness to quit.

**Results:**

Follow-up rates at day 1 were 93% (56/60) for the TG and 100% (60/60) for the CG. At postintervention, the TG reported 23% (14/60) abstinence on that day compared with 5% (3/60) in the CG. This difference was statistically significant (*χ*^2^_1_=8.3; *P*=.004). The TG reported sustained abstinence of 33% (20/60) at 90 days. Since only 20% (12/60) of participants in the CG completed the 90-day follow-up, we did not conduct a statistical comparison between groups at this follow-up time point. Among participants still smoking at postintervention, the TG was significantly more ready to quit compared to the CG (TG: mean 7.71, SD 0.13; CG: mean 7.16, SD 0.13; *P*=.005). A total of 41% (23/56) of participants completed the treatment in the time frame recommended by the program.

**Conclusions:**

Results provide initial support for participant adherence to and efficacy of Mindcotine and warrant testing the intervention in a fully powered randomized trial. However, feasibility of trial follow-up assessment procedures for control group participants needs to be improved. Further research is needed on the impact of VR-MET on long-term outcomes.

**Trial Registration:**

ISRCTN Registry ISRCTN50586181; http://www.isrctn.com/ISRCTN50586181

## Introduction

### Background

Tobacco use remains one of the biggest threats to public health and the leading preventable cause of mortality and morbidity worldwide [1]. Interventions on web-based platforms and mobile apps, among them smoking cessation apps, can deliver effective interventions for various diseases and behavioral disorders [2-16]. Smartphone-based smoking cessation apps can provide an important channel for offering interventions to the entire population [17]. However, participant adherence remains a challenge, and many of these apps struggle to maintain high levels of adherence, thus limiting their potential effectiveness [18].

In addition to smartphone-based interventions for smoking cessation, there have been promising developments in the use of virtual reality (VR). VR is defined as “real-time interactive graphics with [3-dimensional] models, combined with a display technology that gives the user the immersion in the model world and direct manipulation” [19]. The use of VR in digital medicine has been applied in consultancy and hospitals and accompanied by a health professional [20]. It has been demonstrated that the introduction of VR into treatment can improve patient engagement in a range of chronic disease interventions [21]. However, such technology has never been tested for tobacco use disorder as a self-assisted and remote solution to take advantage of the knowledge on how virtual environments can be used to elicit and reduce cravings and support smoking cessation.

Cravings are defined as intense urges or impulses for substance use [22] and are an essential component of substance use disorders, as they provoke drug-seeking behavior [23]. External cues can provoke cue-induced cravings [24] and thus provoke relapse [25,26]. Taken together, these findings suggest that craving is a critical target in the development of novel therapeutics for tobacco use disorder treatment [27,28].

Cue exposure therapy for substance use disorders involves controlled and repeated exposure to drug-related cues in order to extinguish cue-induced cravings [29], and VR technology is a potential mode of cue presentation [30,31]. Virtual reality exposure therapy (VRET) has already been used for smoking cessation [32,33], and studies have applied rigorous systematizations in VRET and cognitive behavioral therapy (CBT) [34-37], providing further evidence that this technique can reduce craving and smoking behavior, with similar effectiveness as stand-alone CBT.

Mindfulness is usually thought of as the awareness to attend to any thought, feeling, or sensation that occurs by simply acknowledging it, without attempting to regulate emotions [38]. Mindfulness training is already used in mobile health (mHealth) smoking cessation interventions [39] by teaching individuals to pay attention to the present moment, understand affective states and cravings to smoke as they appear, and consciously choose to let them pass without impulsively reacting to them. Preliminary evidence suggests that mindfulness-based interventions are associated with increased efficacy compared with other smoking cessation treatments [40,41]. Despite these promising results, in-person mindfulness training continues to present certain challenges. It requires experienced psychotherapists, increases time demands, limits access, and elevates costs [39].

### An Innovative Intervention: Mindcotine

The aim of the current study was to develop an accessible and cost-effective digital intervention for smoking cessation that uses the latest technology adapted for large-scale use and to evaluate participant adherence and smoking cessation outcomes. Our novel intervention combines exposure to smoking-related cues in ecological situations using virtual reality and mindfulness as a tool to cope with in situ cravings, bodily sensations, affective states, and automatic reactions. The merge of these psychological frameworks are expected to work synergistically by reducing cravings. On the one hand, cue exposure treatment involves repeated exposure to smoking-related stimuli in order to elicit and, over time, extinguish cue-induced cravings. In addition, mindfulness techniques work on top of the immersive experience by helping the user to focus on cravings in the present moment. Mindfulness practice includes elements of acceptance and compassion through a perspective of curiosity towards the emerging sensations. Taken together, this intervention can help the user stay grounded in the present moment while acknowledging internal and external triggers. Moreover, these virtual reality and mindfulness sessions are part of a classic cognitive behavioral smoking cessation program that provides information on relevant topics daily through CBT notifications and community support among users moderated by psychologists and mindfulness facilitators. We hypothesized that this novel intervention approach would increase both adherence and abstinence rates among participating smokers compared to a treatment-as-usual control group.

## Methods

### Research Design

The design of the study follows the recommendations for clinical trials in health using virtual reality, in particular, tier VR2 [42], focusing on acceptability, feasibility, tolerability, and initial clinical efficacy. We conducted a clinical trial with a control group, including baseline and follow-up assessments at days 1 and 90 posttreatment.

### Procedure

The study was conducted from February to April of 2018 with a group of smokers from Buenos Aires, Argentina. The administration to the treatment group was carried out through an onboarding process that established the framework of the experience and the basic principles of the use of VR. The treatment was remotely self-administered through the use of a mobile app. The control group received a smoking cessation manual of the Argentine Ministry of Health.

**Figure 1:**
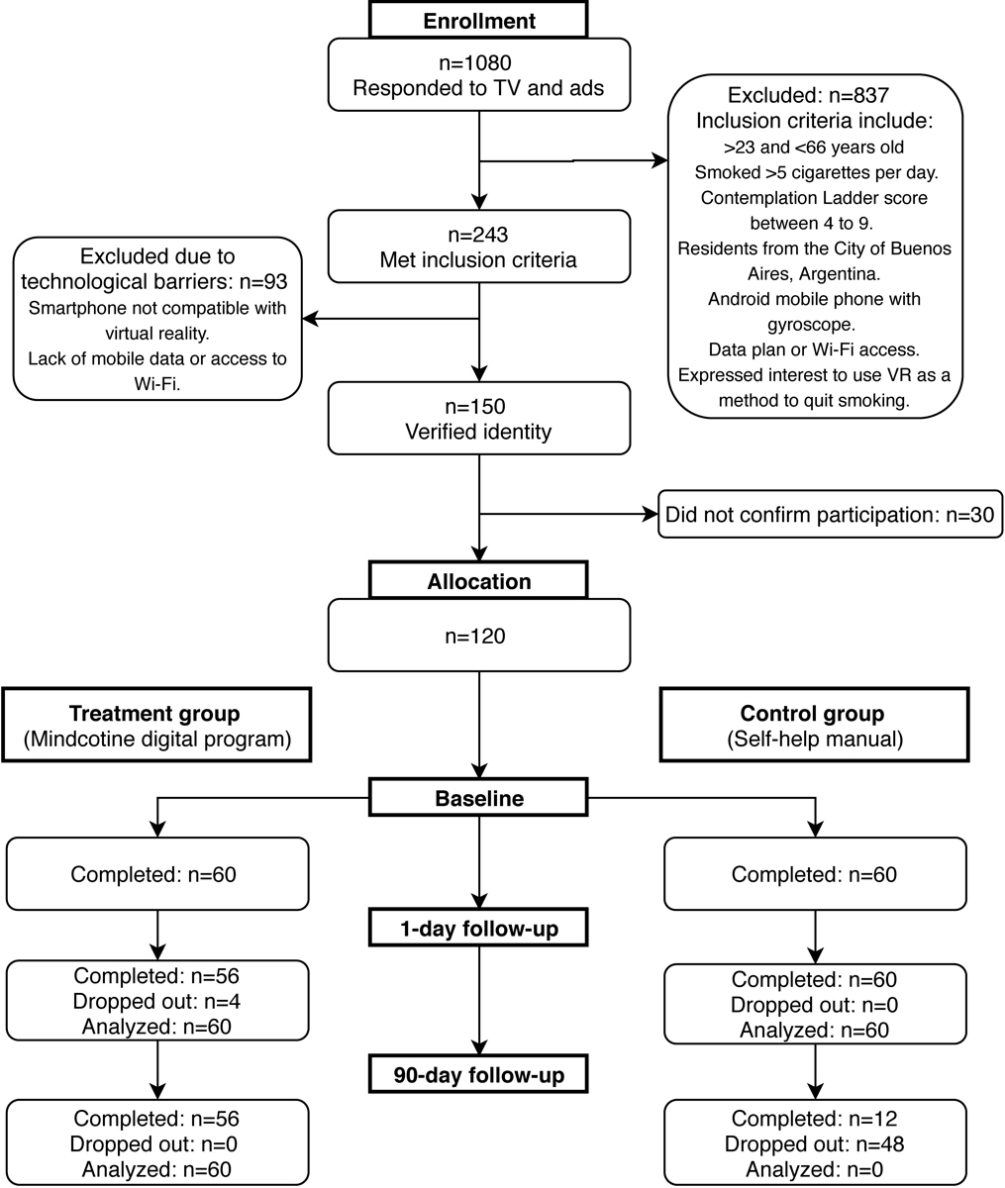
Participant flow through the Mindcotine method smoking cessation trial. TV: television; VR: virtual reality.

The intervention group completed the onboarding process, which included digital informed consent, and received an intervention kit, which included guidelines that explained the use of the program, a unique ID to access the app, one cardboard headset, three stickers to announce smoke-free areas, and two wrist bracelets as a behavior replacement method that smokers could use to snap on their wrists when faced with a craving to smoke. Participants were also instructed to download the mobile app from the Google Play Store (Google Corp). The participants were trained in assembling the cardboard headset (see Multimedia Appendix 1) and app usability, and they completed the baseline assessment and received an explanation of the activities to be carried out during the 21-day intervention phase. The control group signed a digital informed consent sent to their emails and received the online version of the smoking cessation manual developed by the Office of the President of the Argentine Nation [43]. Intervention group participants received the first follow-up 1 day after they completed all 21 days of intervention content, which for some participants took longer than 21 days (postintervention follow-up) and another follow-up assessment at day 90 of posttreatment. Control group participants were assessed at days 30 and 90 after they had received the smoking cessation manual. All assessment invitations were sent via email and assessments were completed online using the Typeform (Typeform SL) platform. Figure 1 shows the participant flow diagram of the study. Participants did not receive any incentives. All study procedures were approved by the Institutional Review Board of the University of Flores, Buenos Aires, Argentina. The trial was registered in the International Standard Randomized Controlled Trial Number Registry (50586181).

### Participants

In order to be included in the study, the selected participants had to meet the following criteria: (1) be aged between 24 and 65 years, representing smokers with a high prevalence of daily smoking [44]; (2) consume a minimum of 5 cigarettes per day, with a score of 4 to 9 on the Contemplation Ladder [45]; (3) be residents in the city of Buenos Aires; (4) own an Android mobile phone with gyroscope; (5) have a data plan or Wi-Fi access; and (6) have an interest in using VR as a method to quit smoking. Each of these criteria was based on previous work with mobile apps for smoking cessation [46]. The one difference in our inclusion criteria compared with this existing research was that we also included smokers that scored below 7 on the Contemplation Ladder in order to investigate intervention effects for smokers not motivated to quit. Participants were excluded if they were diagnosed with a current psychiatric disorder.

For the recruitment of treatment and control group participants, unpaid advertisements were posted for 75 days on the Mindcotine page on Facebook, and a 13-minute segment was aired on Argentine national public television (channel C5N). The social network advertisements linked invitations directly to a screening questionnaire on the Typeform platform [47], which was completed by 1080 potentially eligible volunteers from all over Argentina. Of these initial applicants, 234 subjects lived in the city of Buenos Aires and fulfilled all other necessary inclusion criteria, and telephone contact was established to verify the data. A total of 150 participants were accepted after establishing contact and verifying their responses. However, 30 of these individuals did not confirm their participation, which resulted in a final sample of 120 volunteers. Participants were randomized into the treatment group (TG) (n=60) or control group (CG) (n=60) 1:1 using a blocked random assignment sequence. The treatment group was invited to the research site (University of Flores) and all participants signed an informed consent form, underwent the face-to-face onboarding process with one member of the study team, and were given the Mindcotine Kit to begin the study. The control group was contacted through email, provided digital informed consent, and received the online version of the smoking cessation manual by the Argentine Ministry of Health.

### Intervention

**Figure 2:**
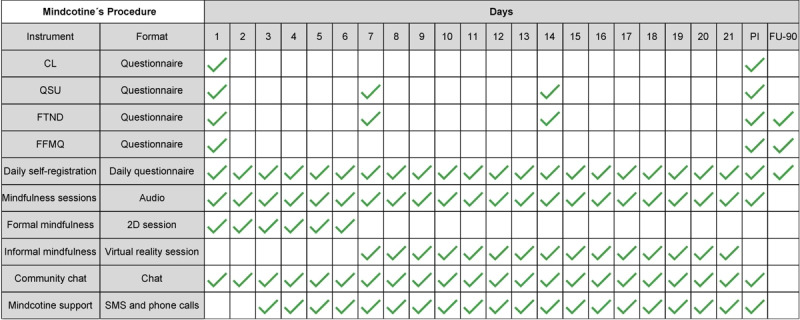
Mindcotine procedure flow diagram. 2D: 2-dimensional; CL: Contemplation Ladder; FFMQ: Five Facet Mindfulness Questionnaire; FTND: Fagerström Test of Nicotine Dependence; FU-90: 90-day follow-up; PI: postintervention; QSU: Questionnaire for Smoking Urges.

The app consists of a 21-day treatment that includes 2 main activities each day, which become available after completing the activities of the previous day (see Figure 2). Intervention development was conducted iteratively using face-to-face testing and focus groups, with feedback from 250 smokers who tested the virtual reality environment of the intervention. Moreover, we reviewed existing smoking cessation apps and features related to trigger identification and reflective questions.

The elements of the program were (1) practice sessions in formal mindfulness, (2) practice sessions in informal mindfulness using virtual reality mindful exposure therapy (VR-MET), (3) daily self-reports, (4) peer-to-peer support, and (5) Mindcotine support.

First, practice sessions in formal mindfulness included 6 sessions of mindfulness in video format of up to 10 minutes each and 7 sessions in audio format of 3 to 10 minutes each. These were based on the works of Kabat-Zinn [48] and Bowen and Marlatt [40], where the user is given an initial introduction to mindfulness involving the recognition of bodily sensations and the ability to practice nonreactivity to emotions and thoughts related to smoking, from a compassionate and nonjudgmental position.

Second, practice sessions in informal mindfulness using VR included 2 sessions of VR-MET, each lasting 10 minutes. These sessions included a selection of 2 virtual environments that combined the awareness of the act of smoking and the recognition of craving from a perspective of acceptance and commitment. This selection is the result of an adaptation of the previously conducted work synthesized by Brewer [49] and included virtual environments used in cue exposure therapy. Regarding VR-MET design, each of the virtual environments was created based on previous research that proved the environment to elicit craving [50] and was recorded with an Insta360Pro camera (Insta360) [51] in locations in Mexico City, Mexico, and Buenos Aires, Argentina. The animated parts of the environments were created in Unreal Engine 4 (Epic Games) and based on previous research to induce emotional states of tranquility and relaxation [52] (see Multimedia Appendix 2). The mindfulness audio from both environments was chosen to work consciously with craving-related acts (“RAIN”: Recognize, Accept, Investigate and Nourish, and “Act of Smoking”: consciously review each moment of the act itself). Each video was repeated over a period of 14 consecutive days, based on previous research regarding the time of exposure in virtual environments and the time between the exposures [30,34,37,53].

Third, daily self-reports were included. At the end of the day, each user reported on the app their total daily number of cigarettes, reasons that any cravings were triggered, and a written answer to the question “What do you think has changed in your relationship to smoking as of today?” (nightly reflections). This data was collected on the Typeform platform embedded in the app.

Fourth, peer-to-peer support was provided via the app’s group chat feature for interacting with all other participants. The group chat was moderated by a psychologist and a mindfulness facilitator to promote engagement and respond to participant questions.

Fifth, Mindcotine support could be prompted. If participants were inactive for a certain amount of time, they received a text message (after 2 days) and a phone call (after 4 days) to encourage engagement within the program. Participants could contact Mindcotine for technical support by email anytime as well.

**Figure 3:**
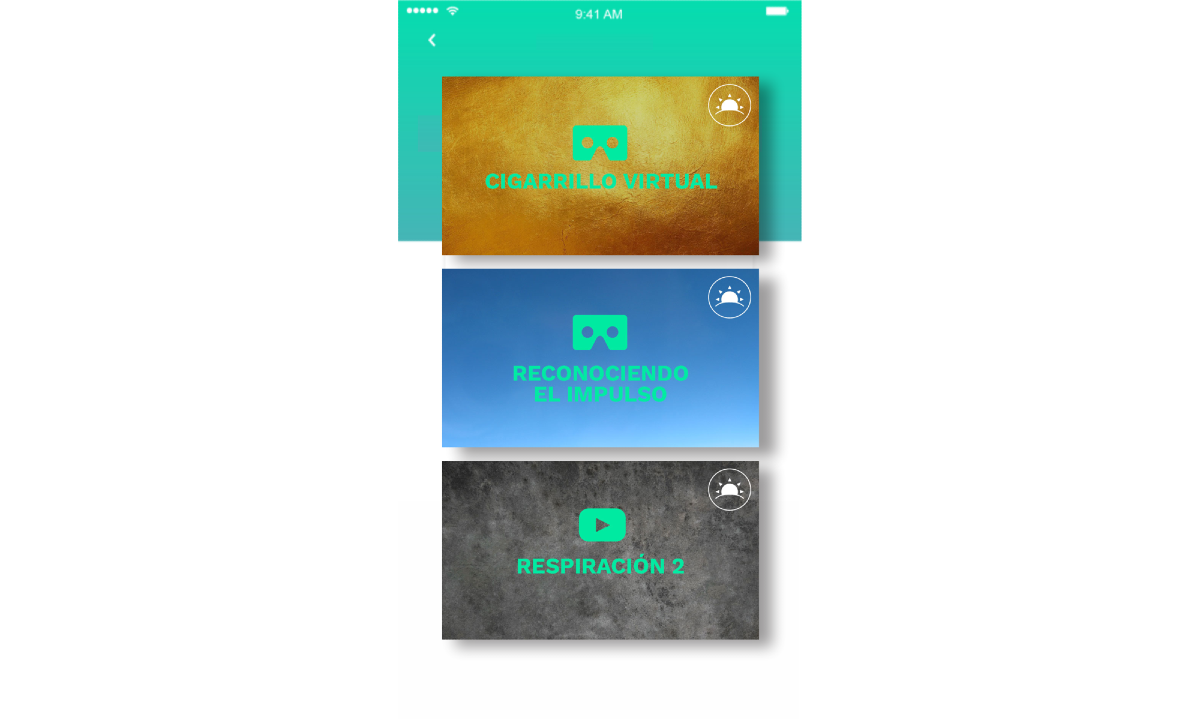
App screenshot of the home screen showing mindfulness activities.

**Figure 4:**
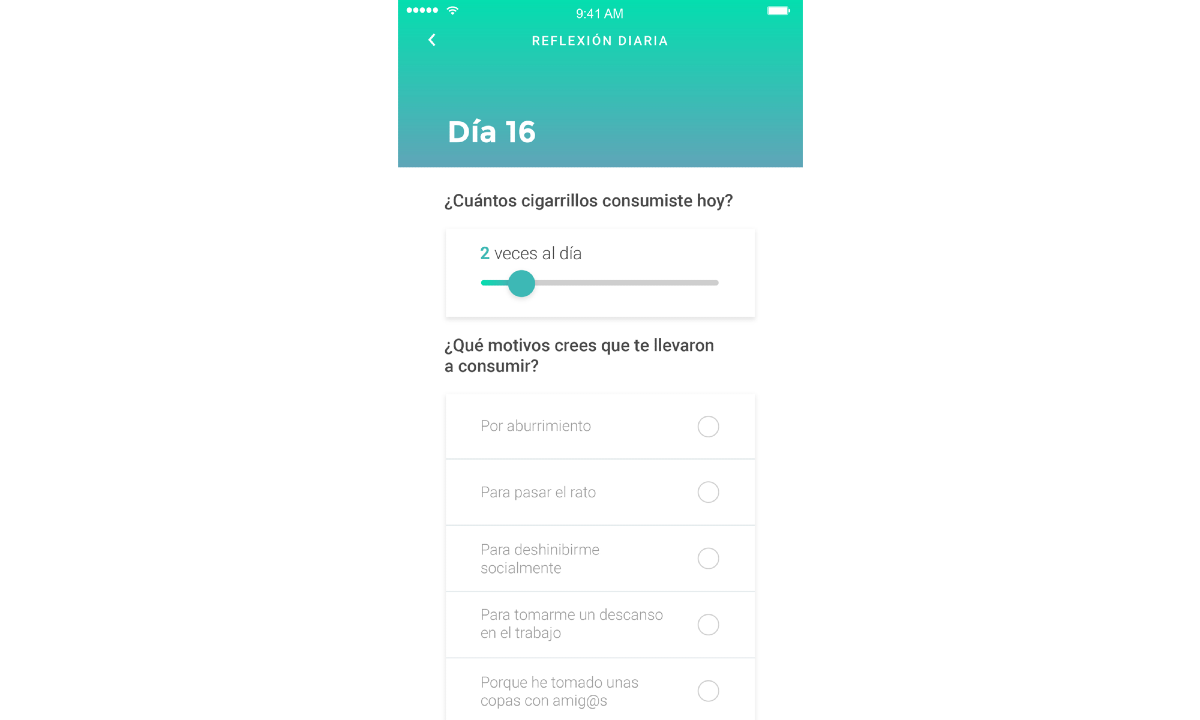
App screenshot of the nightly reflection.

**Figure 5:**
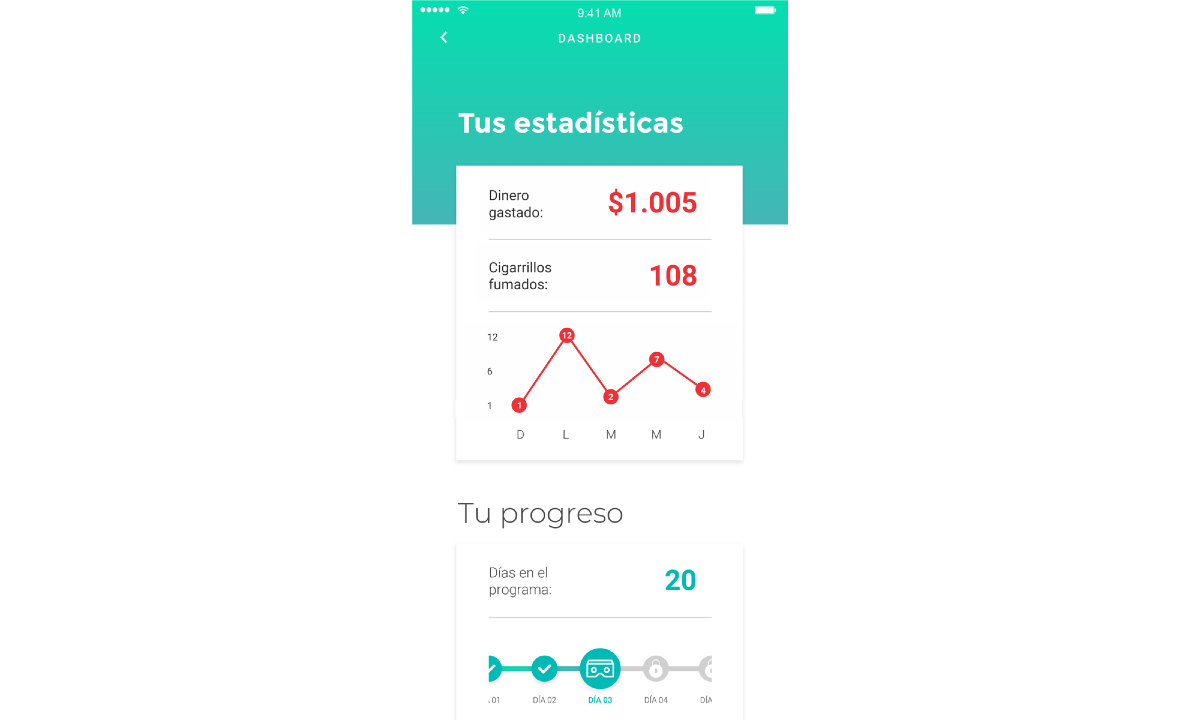
App screenshot on the dashboard showing statistics about money and the number of smoked cigarettes.

**Figure 6:**
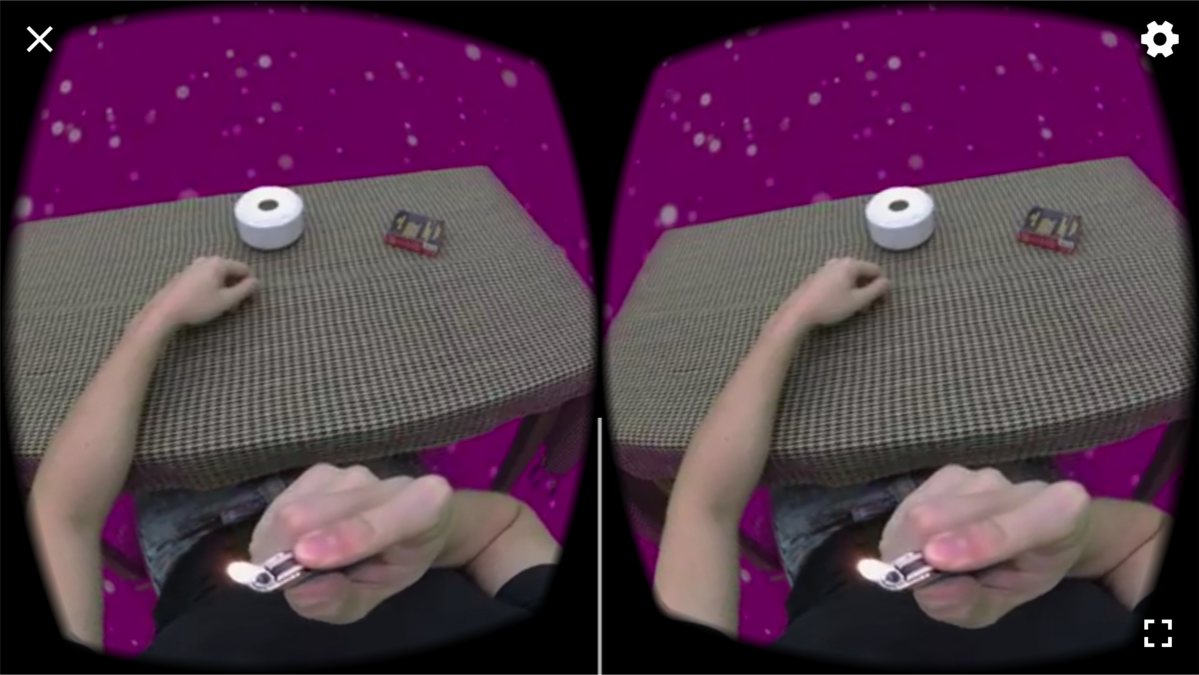
Virtual reality mindful exposure therapy screenshot: Act of Smoking.

These components can be seen in Figure 3 (mindfulness exercises), Figure 4 (nightly reflection), Figure 5 (dashboard), and Figure 6 (VR-MET).

### Measures

#### Baseline Assessment

The baseline assessment was conducted on the app and included demographic data (age, gender, education), smoking behavior (number of cigarettes per day, years of smoking, previous cessation attempts, readiness to quit), characteristics of the environment and family context (marital status, children, living with smokers), and other health-related characteristics (body mass index, physical activity, previous meditation experience).

#### Primary Outcome

The primary outcome was self-reported abstinence at postintervention, assessed 1 day after the end of the program. The question used for assessment was “Did you smoke tobacco in the last 24 hours?”

#### Secondary Outcomes

##### Sustained Abstinence

Sustained abstinence was self-reported abstinence at 90 days after the end of the program in the TG. The question used for assessment was “Did you smoke tobacco in the last 90 days?”

##### Adherence

Adherence was assessed using log file data collected by employing the platform Amplitude (Amplitude Inc) [54], which measured total use of the app for each participant. Adherence to treatment was categorized as the percentage of participants who completed all daily activities and all daily diary questionnaires throughout the intervention with intervals of discontinuity no greater than 4 consecutive days. We operationalized full adherence as to whether or not the participant completed the treatment in the suggested time (ie, 21 days without any breaks), regular adherence as whether participants completed all daily activities in up to 60 days, and depth of adherence as the total number of mindfulness training minutes [55]. Chat room activity beyond the initial onboarding post was measured by the number of participant comments in the chat room and dichotomized to any activity versus no activity.

##### Cigarette Consumption

Participants were instructed to report the number of cigarettes they smoked each day during treatment as part of their daily self-reports, at postintervention, and at 90-day follow-up. Over the treatment period, the daily number of cigarettes was stored and averaged per week.

##### Craving

Craving was assessed using the Questionnaire for Smoking Urges (QSU) [56], which consists of a 7-point Likert scale. These data were collected through an online survey at baseline, at the end of days 7 and 14, and at postintervention. The internal consistency for the overall scale was adequate (α=.87).

##### Mindfulness

Mindfulness was assessed using the Five Facet Mindfulness Questionnaire (FFMQ) [57]. The 5 dimensions are observing, describing, acting with awareness, not judging internal experience, and not reacting to internal experience. These data were collected through an online survey at baseline, at postintervention, and at 90-day follow-up.

##### Readiness to Quit

Readiness to quit was assessed using the Contemplation Ladder [45], which consists of 11 rungs and 5 anchor statements reflecting the stages of change and was designed to measure readiness to quit smoking. It was assessed through an online survey at baseline and at postintervention.

##### Nicotine Dependence

Nicotine dependence was assessed using the Fagerström Test of nicotine dependence [58], which consists of a 6-item self-report scale and observes responses suggestive of physiological dependence on nicotine. These data were collected through an online survey at the beginning of the program, at the end of day 7, day 14, postintervention, and 90-day follow-up. The internal consistency for the overall scale was adequate (ɑ=.81).

#### Data Analysis

Statistical analysis was performed with SPSS for Windows (version 22; IBM Corp). Abstinence rates between groups were compared using a chi-square test. Participants with missing data at follow-up were assumed to be smoking. Repeated measure analyses of variance (ANOVAs) were used to determine between-group differences and intervention effects for continuous variables. The group factor had 2 levels, corresponding to intervention and control group, and the time factor had 3 levels, corresponding to the 3 assessment points (baseline, postintervention, and 90-day follow-up). Post hoc analyses were conducted to determine significant pairwise comparisons. ANOVAs and post hoc tests were conducted to determine changes within the treatment group over time. All *t* tests in the study were 2-tailed.

## Results

### Recruitment and Participation in the Intervention

Participant baseline characteristics are displayed in Table 1. On average, participants in the sample were 43.25 years old (SD 10.06) and 47.5% (57/120) were female. The largest group in terms of completed educational level was high school (45/120, 37.5%), followed by university (43/120, 35.8%). On average, the sample started smoking 19.15 years ago (SD 12.35) and consumed an average of 10.77 (SD 5.47) cigarettes per day. Of all participants, 14.2% (17/120) lived with other smokers in their homes. The entire sample reported a moderated nicotine dependence index, based on the mean scores obtained in the Fagerström Nicotine Dependence Test [58], of 4.48 (SD 1.55). Only 40.8% (49/120) had practiced meditation at least once in their life. No statistically significant differences were observed between treatment and control group on any variables at baseline.

**Table 1:** Participant baseline characteristics (N=120).

Characteristics			Full sample (N=120)		Treatment group (n=60)		Control group (n=60)
Age (years), mean (SD)			43.25 (10.06)		44.06 (9.89)		42.44 (10.23)
Gender (female), n (%)			57 (48)		33 (55)		24 (41)
**Education, n (%)**							
	PhD or higher	9 (8)		7 (11)		2 (5)	
	University	43 (36)		25 (38)		18 (30)	
	Undergraduate	10 (8)		3 (3)		7 (6)	
	Technical	6 (5)		3 (3)		3 (8)	
	High school	45 (38)		19 (28)		26 (44)	
	Primary school	7 (6)		3 (5)		4 (6)	
Years since started smoking, mean (SD)			19.15 (12.35)		19.40 (12.28)		18.91 (12.42)
Current cigarette consumption (cigarettes per day), mean (SD)			10.77 (5.47)		11.09 (5.27)		10.45 (5.67)
At least 1 attempt to quit in the last year, n (%)			65 (54)		32 (53)		33 (55)
Live with other smokers, n (%)			17 (14)		10 (17)		7 (12)
Nicotine dependence (FTND^a^), mean (SD)			4.48 (1.55)		4.22 (1.51)		4.75 (1.56)
Craving (QSU^b^), mean (SD)			29.5 (9.67)		30.28 (11.72)		28.71 (7.08)
**Readiness to quit (Contemplation Ladder), mean (SD)**			6.68 (1.17)		6.53 (1.14)		6.83 (1.18)
	Precontemplation, n (%)	5 (4)		2 (3)		3 (5)	
	Contemplation, n (%)	45 (38)		26 (43)		19 (32)	
	Preparation, n (%)	67 (56)		31 (52)		36 (60)	
	Action, n (%)	3 (3)		1 (2)		2 (3)	
No experience in meditation, n (%)			71 (59)		33 (54)		38 (64)
Five Facet Mindfulness Questionnaire, mean (SD)			123.16 (8.73)		123.94 (9.55)		122.43 (7.83)

^a^FTND: Fagerström Test of Nicotine Dependence.

^b^QSU: Questionnaire for Smoking Urges.

### Primary Outcomes

At postintervention, the TG reported 23% (14/60) abstinence on that day compared with 5% (3/60) of the CG. This difference was statistically significant (*χ^2^*_1_=8.3; *P*=.004).

### Secondary Outcomes

#### Sustained Abstinence

The TG reported 33% (20/60) sustained abstinence on the 90-day follow-up, compared with 5% (3/60) of participants in the CG. Since only 20% (12/60) of participants in the CG completed the 90-day follow-up, we did not conduct a statistical comparison between groups at this follow-up time point.

#### Adherence Rates

Intervention adherence was analyzed only in the TG, and 93% (56/60) of participants finished the 21-day program. Of those who finished, 41% (23/56) were fully adherent to the program (ie, completed all daily sessions and nightly reflections 21 days in a row) and 59% (33/56) were regularly adherent (ie, completed the program in >21 days), completing the program in 28.56 days on average. At the postintervention, 30% (7/23) of fully adherent participants reported smoking abstinence, while only 21% (7/33) of regularly adherent participants were abstinent. At the 90-day follow-up, smoking abstinence rates were 39% (9/23) among fully adherent participants and 33% (11/33) among regularly adherent participants. A statistically significant difference in readiness to quit was found between the fully and the regularly adherent group at baseline (t_55_=3.092; *P*=.003), with fully adherent participants reporting greater readiness to quit. No differences were found regarding nicotine dependence (t_55_=1.206; *P*=.23) at baseline, nor regarding abstinence rates between the 2 adherence groups at postintervention (*χ^2^*_1_=6.1; *P*=.43) and at 90-day follow-up (*χ^2^*_1_=1.9; *P*=.66)

If participants had not engaged with the program for 2 subsequent days, they were contacted in order to improve engagement. A total of 92% (54/60) of participants were contacted through SMS text messaging once; 65% (39/60), twice; 53% (32/60), thrice; and 34% (20/60) were contacted through both SMS text messaging and phone call reminders. Depth of adherence for all participants, measured by the number of minutes of mindfulness training during the program (including virtual reality, video format, and audio format), was 259.05 minutes on average, with a maximum of 386 minutes and a minimum of 216 minutes.

For those who reported abstinence at postintervention, the average mindfulness minutes trained was 250.43 minutes, while the average was 261.93 minutes among those who continued smoking. Statistically significant differences were not found between groups (t_55_=–1.291; *P*=.20).

Participant activity in the chat room beyond an initial introduction message suggested in the onboarding process was low and only 13 participants commented in the chat room beyond the onboarding. Chat room activity was not associated with smoking cessation outcomes, having only 1 participant that successfully quit smoking while being active in the chat.

#### Cigarette Consumption

Statistically significant differences in cigarettes per day over time were found between the TG and the CG (*F*_5,114_=95.73; *P*<.001) in the third week of the intervention and at postintervention. At intervention week 3, the TG consumed significantly fewer cigarettes than the CG (TG: mean 6.92, SD 5.26; CG: mean 9.03, SD 5.42; *P*=.03). Significant differences in cigarettes per day between groups were also found at postintervention (TG: mean 5.07, SD 5.65; CG: mean 9.53, SD 0.56; *P*<.001).

#### Readiness to Quit

The means comparison in the Contemplation Ladder for the TG and the CG showed statistically significant differences between groups (*F*_2,113_=4.55; *P*=.01). Post hoc comparisons revealed that there were no differences at baseline (TG: mean 6.53, SD 0.14; CG: mean 6.83, SD 0.18; *P*=.32), but there were at postintervention (TG: mean 7.71, SD 0.13; CG: mean 7.16, SD 0.13; *P*=.005).

### Mean Differences Within the Treatment Group Over Time

#### Cigarette Consumption

Post hoc tests after ANOVA were conducted for cigarette consumption, and statistically significant differences over time were found within the TG (*F*_4,52_=13.79; *P*<.001). Participants significantly reduced their cigarettes per day from baseline (mean 11.09, SD 5.27) to postintervention (mean 6.05, SD 5.67; *P*<.001), as well as from baseline to 90-day follow-up (mean 5.07, SD 5.65; *P*<.001).

#### Mindfulness

Moreover, according to paired *t* test comparisons for FFMQ scores, no statistically significant difference was found between baseline (mean 123.95, SD 9.55) and postintervention (mean 122.61, SD 6.31; t_55_=0.772; *P*=.44). However, FFMQ at 90-day follow-up (mean 115.89, SD 12.30) was significantly lower than scores at baseline (t_55_=5.594; *P*<.001) and postintervention (t_55_=3.234; *P*=.002).

#### Craving

A significant reduction in self-reported craving over time was observed in the treatment group (baseline: mean 30.28, SD 11.72; intervention week 1: mean 30.01, SD 11.75; intervention week 2: mean 28.00, SD 13.00; postintervention: mean 26.00, SD 11.37). These differences over time were statistically significant (*F*_1,24_=3.725; *P*=.005).

## Discussion

### Principal Findings

This is the first study to report results of a pilot trial testing a VR smartphone-based smoking cessation program using remote and self-assisted delivery. At postintervention, the intervention group had a significantly higher abstinence rate (14/60, 23% abstinence) compared with the control group, which received a smoking cessation manual (3/60, 5% abstinence). Moreover, the intervention resulted in a 33% (20/60) abstinence rate at 90-day follow-up and high levels of adherence and engagement. Findings suggest the potential efficacy of a smartphone-based VR intervention that combines exposure therapy and mindfulness for smoking cessation.

When compared with other smoking cessation app studies, abstinence rates observed in the current study were in a similar range. For instance, an 8-week single-arm trial of a smartphone app that delivered essential features of US clinical practice guidelines with personalization resulted in a 26% abstinence rate at 30-day follow-up [59]. Another single-arm trial based on acceptance and commitment therapy had abstinence rates of 33% at 7-day follow-up and 28% at 30-day follow-up [60]. These rates are similar to the 23% (14/60) and 33% (20/60) observed in our study and provide confidence for further development and testing.

With 93% (56/60) of participants finishing the 21-day program, the current study had a high completion and low dropout rate. In line with other studies, we used several strategies to enhance adherence, including SMS text messaging and phone call reminders [55]. Consistent with our findings, adherence rates were also above 80% in the above-mentioned 8-week single-arm clinical trial [59] and in a randomized controlled trial of a text messaging program [61]. Of all 56 TG participants who finished the program, 23 completed without any gaps in treatment adherence and were classified as fully adherent, while the other 33 participants took an average of 28.56 days to complete the program and were classified as regularly adherent. The fully adherent group reported greater readiness to quit at baseline. Abstinence at postintervention and 90-day follow-up was higher among fully adherent participants compared with regularly adherent participants. These findings suggest that repeated exposure to smoking-related cues in virtual environments alongside mindfulness practice on a consecutive daily basis may increase abstinence outcomes compared with sessions that are more spaced out over time. Thus, a more consistent and disciplined training using virtual reality mindfulness-based exposure therapy to both internal and external triggers over a shorter period of time may result in better outcomes. Based on other studies [62], the chat room activity was low probably due to the lack of promotion to increase engagement through tailored material by the moderators. Overall, the methods used to strengthen engagement have been shown to have value and can be further developed.

Our results also indicate that mindfulness scores measured by the FFMQ significantly decreased within the TG from baseline to 90-day follow-up. It is unclear what may have caused this decrease despite the promising smoking cessation outcomes of our intervention. Given that the current study is the first to combine virtual reality exposure therapy and mindfulness-based relapse prevention for smoking cessation, replication of these findings is needed. Moreover, future studies should further explore the mechanisms of action of this novel intervention approach.

Other studies using VR and exposure therapy in smoking cessation showed similar results in decreasing cue-induced craving, such as a randomized clinical trial that combined CBT and virtual reality cue exposure therapy (VR-CET), which found a significant reduction of cue-induced cravings after group-based sessions over the course of 6 months with 5 individual sessions of VR-CET [37]. In the current study, the virtual reality environments exposed participants to smoking-related cues and at the same time presented a mindfulness narrative based on relapse prevention. Therefore, the intervention simultaneously elicits cravings by means of VRET and provides the user with tools for reducing these cravings by means of mindfulness. Our results of QSU craving scores over time show that scores did not decrease during the first week of treatment, in which there was no exposure therapy. However, self-reported cravings decreased during the following 14 days of the program, in which VR-MET was delivered.

Feasibility of conducting the trial was demonstrated by the fact that we were able to recruit 120 participants in the current pilot trial. However, feasibility of collecting follow-up data from a control group needs to be improved. The delivery of the intervention was feasible and acceptable to participants; 41% (23/56) of intervention participants completed the entire intervention content in 21 days, as recommended, and the average adherence to mindfulness training was 259.05 minutes per participant. Feasibility of the VR component as a central element of the program was high, with all participants practicing at least 15 informal mindfulness practice sessions lasting 10 minutes each. Even though this intervention introduced VR and mindfulness training to a population that had almost never tried any of these approaches before, the intervention had high engagement rates, suggesting remote and self-administered VR can be used as a strategy for improving adherence to mHealth interventions. These findings are in line with those of existing studies, which have shown that VR can enhance treatment fidelity by having behavioral interventions delivered by a programmed avatar [21] and that VR environments offer existing opportunities to enhance a patient´s involvement in treatments [63].

Given that VR in mental health to date has predominantly been used in inpatient hospital environments [20], the recruitment method used in the current study to test the potential of VR as a self-administered and remote smoking cessation intervention on an outpatient population is worth mentioning. After appearing on national television, more than 1000 volunteers from all over Argentina registered within a few hours. The average age of these volunteers was 41 years, which was considerably older than we had anticipated. The fact that we were able to recruit this population for a smartphone-based smoking cessation study using a VR cardboard headset demonstrates that this intervention can be accessible to populations that do not need to be exceptionally tech savvy. In light of existing challenges to recruit participants for mHealth interventions [64], this particular recruitment strategy may prove valuable for future research.

The creation of Mindcotine involved a group of psychologists, psychiatrists, and other physicians, as well as developers, actors, and mindfulness facilitators, and took over 6 months. While initial development costs were high, the fact that this program can be delivered remotely and self-administered makes it a low-cost and accessible intervention to promote smoking cessation.

### Limitations

The current study has several limitations. The follow-up time was relatively short, given that only between 3% and 5% of smokers remain abstinent within the first year of quitting [65]. Follow-up assessment time points were not identical across intervention and control groups. Before the current pilot trial, we did not know how long participants would take to complete the entire program, thus we selected a 30-day follow-up time point for the control group. Future research will adhere to a consistent follow-up assessment time point across intervention and control groups. Completion of the 90-day follow-up in the control group was low. It is possible that control group participants may not have been motivated to complete follow-up surveys due to not seeing changes in their behavior, not receiving a digital intervention, or not receiving any monetary incentive. Control group follow-up rates need to be improved in future trials of this intervention. Other limitations include that at baseline, participants were not regular users of VR, the virtual intervention content was not interactive, only an Android version of the intervention app was available, and the version of the intervention tested in the current study did not include features for the users to track cravings on the app and in virtual reality environments. These features are currently in development for future versions of the intervention. Moreover, abstinence at follow-up was self-reported and subsequent investigations should include biochemical verification of outcomes.

Finally, the face-to-face meeting and the onboarding process at the beginning of the program could have impacted the high adherence rates observed in this trial and online onboarding may work differently. Future studies should administer the entire program remotely.

### Conclusion

Overall, our VR smartphone-based Mindcotine intervention to support smoking cessation showed great potential with regard to participant adherence and initial efficacy. These findings warrant testing the intervention in a fully powered randomized trial including longer follow-up intervals to investigate relapse prevention and biochemical verification of abstinence.

## Appendix

Multimedia Appendix 1 Instructional video. How to assemble the virtual reality headset.

Multimedia Appendix 2 Brief VR-MET environment.

Multimedia Appendix 3 CONSORT-eHEALTH checklist (V 1.6.1).

## Abbreviations

ANOVA: analysis of variance
CBT: cognitive behavioral therapy
CG: control group
FFMQ: Five Facet Mindfulness Questionnaire
mHealth: mobile health
QSU: Questionnaire for Smoking Urges
TG: treatment group
VR: virtual reality
VR-CET: virtual reality cue exposure therapy
VRET: virtual reality exposure therapy
VR-MET: virtual reality mindful exposure therapy
