# Supplementary material for: Virtual Reality Smartphone-Based Intervention for Smoking Cessation: Pilot Randomized Controlled Trial on Initial Clinical Efficacy and Adherence
Source: J Med Internet Res. 2020 Jul 29;22(7):e17571. doi: 10.2196/17571 (PMC7424475; doi:10.2196/17571)
Supplement: Multimedia Appendix 3 [file jmir_v22i7e17571_app3.pdf]

CONSORT-EHEALTH (V 1.6.1) - Submission/Publication Form

The CONSORT-EHEALTH checklist is intended for authors of randomized trials evaluating web-based and Internet-based applications/interventions, including mobile interventions, electronic games (incl multiplayer games), social media, certain telehealth applications, and other interactive and/or networked electronic applications. Some of the items (e.g. all subitems under item 5 - description of the intervention) may also be applicable for other study designs.

The goal of the CONSORT EHEALTH checklist and guideline is to be
a) a guide for reporting for authors of RCTs,
b) to form a basis for appraisal of an ehealth trial (in terms of validity)

CONSORT-EHEALTH items/subitems are MANDATORY reporting items for studies published in the Journal of Medical Internet Research and other journals / scientific societies endorsing the checklist.

Items numbered 1., 2., 3., 4a., 4b etc are original CONSORT or CONSORT-NPT (non-pharmacologic treatment) items.
Items with Roman numerals (i., ii, iii, iv etc.) are CONSORT-EHEALTH extensions/clarifications.

As the CONSORT-EHEALTH checklist is still considered in a formative stage, we would ask that you also RATE ON A SCALE OF 1-5 how important/useful you feel each item is FOR THE PURPOSE OF THE CHECKLIST and reporting guideline (optional).

Mandatory reporting items are marked with a red *.
In the textboxes, either copy & paste the relevant sections from your manuscript into this form - please include any quotes from your manuscript in QUOTATION MARKS,
or answer directly by providing additional information not in the manuscript, or elaborating on why the item was not relevant for this study.

YOUR ANSWERS WILL BE PUBLISHED AS A SUPPLEMENTARY FILE TO YOUR PUBLICATION IN JMIR AND ARE CONSIDERED PART OF YOUR PUBLICATION (IF ACCEPTED).
Please fill in these questions diligently. Information will not be copyedited, so please use proper spelling and grammar, use correct capitalization, and avoid abbreviations.

DO NOT FORGET TO SAVE AS PDF _AND_ CLICK THE SUBMIT BUTTON SO YOUR ANSWERS ARE IN OUR DATABASE !!!

Citation Suggestion (if you append the pdf as Appendix we suggest to cite this paper in the caption):
Eysenbach G, CONSORT-EHEALTH Group
CONSORT-EHEALTH: Improving and Standardizing Evaluation Reports of Web-based and

Mobile Health Interventions
J Med Internet Res 2011;13(4):e126
URL: http://www.jmir.org/2011/4/e126/
doi: 10.2196/jmir.1923
PMID: 22209829

* Required

Your name *

First Last

Emilio Goldenhersch

Primary Affiliation (short), City, Country *

University of Toronto, Toronto, Canada

Universidad de Flores, Buenos Aires, Argentina

Your e-mail address *

abc@gmail.com

emiliogolden@gmail.com

Title of your manuscript *

Provide the (draft) title of your manuscript.

Virtual reality smartphone-based smoking cessation: A pilot RCT on initial clinical efficacy and adherence

Name of your App/Software/Intervention *

If there is a short and a long/alternate name, write the short name first and add the long name in brackets.

Mindcotine

Evaluated Version (if any)

e.g. "V1", "Release 2017-03-01", "Version 2.0.27913"

V2, Release 2018-03-14

Language(s) *

What language is the intervention/app in? If multiple languages are available, separate by comma (e.g. "English, French")

Spanish, English

URL of your Intervention Website or App

e.g. a direct link to the mobile app on app in appstore (itunes, Google Play), or URL of the website. If the intervention is a DVD or hardware, you can also link to an Amazon page.

https://play.google.com/store/apps/details?id

URL of an image/screenshot (optional)

Your answer

Accessibility *

Can an enduser access the intervention presently?

- ( ) access is free and open
- ( ) access only for special usergroups, not open
- (•) access is open to everyone, but requires payment/subscription/in-app purchases
- ( ) app/intervention no longer accessible
- ( ) Other:

Primary Medical Indication/Disease/Condition *

e.g. "Stress", "Diabetes", or define the target group in brackets after the condition, e.g. "Autism (Parents of children with)", "Alzheimers (Informal Caregivers of)"

Tobacco Use Disorder

Primary Outcomes measured in trial *

comma-separated list of primary outcomes reported in the trial

Abstinence rate

Secondary/other outcomes

Are there any other outcomes the intervention is expected to affect?

Sustained Abstinence, Adherence rates, Cigarette Consumption, Readiness to quit

Recommended "Dose" *

What do the instructions for users say on how often the app should be used?

- (x) Approximately Daily
- ( ) Approximately Weekly
- ( ) Approximately Monthly
- ( ) Approximately Yearly
- ( ) "as needed"
- ( ) Other:

Approx. Percentage of Users (starters) still using the app as recommended after 3 months *

- (x) unknown / not evaluated
- ( ) 0-10%
- ( ) 11-20%
- ( ) 21-30%
- ( ) 31-40%
- ( ) 41-50%
- ( ) 51-60%
- ( ) 61-70%
- ( ) 71%-80%
- ( ) 81-90%
- ( ) 91-100%
- ( ) Other:

Overall, was the app/intervention effective? *

- [x] yes: all primary outcomes were significantly better in intervention group vs control
- [ ] partly: SOME primary outcomes were significantly better in intervention group vs control
- [ ] no statistically significant difference between control and intervention
- [ ] potentially harmful: control was significantly better than intervention in one or more outcomes
- [ ] inconclusive: more research is needed
- [ ] Other:

Article Preparation Status/Stage *

At which stage in your article preparation are you currently (at the time you fill in this form)

- [ ] not submitted yet - in early draft status
- [ ] not submitted yet - in late draft status, just before submission
- [ ] submitted to a journal but not reviewed yet
- [x] submitted to a journal and after receiving initial reviewer comments
- [ ] submitted to a journal and accepted, but not published yet
- [ ] published
- [ ] Other:

Journal *

If you already know where you will submit this paper (or if it is already submitted), please provide the journal name (if it is not JMIR, provide the journal name under "other")

- ( ) not submitted yet / unclear where I will submit this
- (•) Journal of Medical Internet Research (JMIR)
- ( ) JMIR mHealth and UHealth
- ( ) JMIR Serious Games
- ( ) JMIR Mental Health
- ( ) JMIR Public Health
- ( ) JMIR Formative Research
- ( ) Other JMIR sister journal
- ( ) Other:

Is this a full powered effectiveness trial or a pilot/feasibility trial? *

- (•) Pilot/feasibility
- ( ) Fully powered

Manuscript tracking number *

If this is a JMIR submission, please provide the manuscript tracking number under "other" (The ms tracking number can be found in the submission acknowledgement email, or when you login as author in JMIR. If the paper is already published in JMIR, then the ms tracking number is the four-digit number at the end of the DOI, to be found at the bottom of each published article in JMIR)

- ( ) no ms number (yet) / not (yet) submitted to / published in JMIR
- (•) Other: 17571

TITLE AND ABSTRACT

1a) TITLE: Identification as a randomized trial in the title

1a) Does your paper address CONSORT item 1a? *

I.e does the title contain the phrase "Randomized Controlled Trial"? (if not, explain the reason under "other")

- (selected) yes
- Other:

1a-i) Identify the mode of delivery in the title

Identify the mode of delivery. Preferably use “web-based” and/or “mobile” and/or “electronic game” in the title. Avoid ambiguous terms like “online”, “virtual”, “interactive”. Use “Internet-based” only if Intervention includes non-web-based Internet components (e.g. email), use “computer-based” or “electronic” only if offline products are used. Use “virtual” only in the context of “virtual reality” (3-D worlds). Use “online” only in the context of “online support groups”. Complement or substitute product names with broader terms for the class of products (such as “mobile” or “smart phone” instead of “iphone”), especially if the application runs on different platforms.

	1	2	3	4	5	
subitem not at all important	○	○	○	○	●	essential

Does your paper address subitem 1a-i? *

Copy and paste relevant sections from manuscript title (include quotes in quotation marks "like this" to indicate direct quotes from your manuscript), or elaborate on this item by providing additional information not in the ms, or briefly explain why the item is not applicable/relevant for your study

"Virtual reality smartphone-based"

1a-ii) Non-web-based components or important co-interventions in title

Mention non-web-based components or important co-interventions in title, if any (e.g., “with telephone support”).

	1	2	3	4	5	
subitem not at all important	◉	○	○	○	○	essential

Does your paper address subitem 1a-ii?

Copy and paste relevant sections from manuscript title (include quotes in quotation marks "like this" to indicate direct quotes from your manuscript), or elaborate on this item by providing additional information not in the ms, or briefly explain why the item is not applicable/relevant for your study

Co-interventions such as text and phone calls were made by the Mindcotine team to encourage engagement and adherence to the program. These co-interventions were not directly related to the virtual reality aspect and were not part of the main objective of this study.

1a-iii) Primary condition or target group in the title

Mention primary condition or target group in the title, if any (e.g., “for children with Type I Diabetes”)
Example: A Web-based and Mobile Intervention with Telephone Support for Children with Type I Diabetes: Randomized Controlled Trial

	1	2	3	4	5	
subitem not at all important	○	○	○	○	◉	essential

Does your paper address subitem 1a-iii? *

Copy and paste relevant sections from manuscript title (include quotes in quotation marks "like this" to indicate direct quotes from your manuscript), or elaborate on this item by providing additional information not in the ms, or briefly explain why the item is not applicable/relevant for your study

"smoking cessation"

1b) ABSTRACT: Structured summary of trial design, methods, results, and conclusions

NPT extension: Description of experimental treatment, comparator, care providers, centers, and blinding status.

1b-i) Key features/functionalities/components of the intervention and comparator in the METHODS section of the ABSTRACT

Mention key features/functionalities/components of the intervention and comparator in the abstract. If possible, also mention theories and principles used for designing the site. Keep in mind the needs of systematic reviewers and indexers by including important synonyms. (Note: Only report in the abstract what the main paper is reporting. If this information is missing from the main body of text, consider adding it)

	1	2	3	4	5	
subitem not at all important	○	○	○	○	◉	essential

Does your paper address subitem 1b-i? *

Copy and paste relevant sections from the manuscript abstract (include quotes in quotation marks "like this" to indicate direct quotes from your manuscript), or elaborate on this item by providing additional information not in the ms, or briefly explain why the item is not applicable/relevant for your study

"Participants were randomly assigned to a treatment group (TG), which received a self-assisted 21-day program based on Virtual Reality Mindful Exposure Therapy (VR-MET) sessions, daily surveys, and online peer-to-peer support moderated by psychologists; and a control group (CG), which received the online version of the smoking cessation manual from the Argentine Ministry of Health."

1b-ii) Level of human involvement in the METHODS section of the ABSTRACT

Clarify the level of human involvement in the abstract, e.g., use phrases like “fully automated” vs. “therapist/nurse/care provider/physician-assisted” (mention number and expertise of providers involved, if any). (Note: Only report in the abstract what the main paper is reporting. If this information is missing from the main body of text, consider adding it)

	1	2	3	4	5	
subitem not at all important	○	◉	○	○	○	essential

Does your paper address subitem 1b-ii?

Copy and paste relevant sections from the manuscript abstract (include quotes in quotation marks "like this" to indicate direct quotes from your manuscript), or elaborate on this item by providing additional information not in the ms, or briefly explain why the item is not applicable/relevant for your study

Human involvement had participation during the face-to-face on-boarding session, the intervention as moderators of the peer-to-peer support chat group (1 psychologist and 1 mindfulness facilitator), and when participants had intervals of use longer than 2 days (text message) and 4 days (phone call). The rest was fully automated.

1b-iii) Open vs. closed, web-based (self-assessment) vs. face-to-face assessments in the METHODS section of the ABSTRACT

Mention how participants were recruited (online vs. offline), e.g., from an open access website or from a clinic or a closed online user group (closed usergroup trial), and clarify if this was a purely web-based trial, or there were face-to-face components (as part of the intervention or for assessment). Clearly say if outcomes were self-assessed through questionnaires (as common in web-based trials). Note: In traditional offline trials, an open trial (open-label trial) is a type of clinical trial in which both the researchers and participants know which treatment is being administered. To avoid confusion, use “blinded” or “unblinded” to indicated the level of blinding instead of “open”, as “open” in web-based trials usually refers to “open access” (i.e. participants can self-enrol). (Note: Only report in the abstract what the main paper is reporting. If this information is missing from the main body of text, consider adding it)

	1	2	3	4	5	
subitem not at all important	○	○	○	◉	○	essential

Does your paper address subitem 1b-iii?

Copy and paste relevant sections from the manuscript abstract (include quotes in quotation marks "like this" to indicate direct quotes from your manuscript), or elaborate on this item by providing additional information not in the ms, or briefly explain why the item is not applicable/relevant for your study

"Follow-up assessments were conducted by online survey at post-intervention and 90-day follow-up. The primary outcome was self-reported abstinence at post-intervention, with missing data assumed as still smoking. Secondary outcomes included sustained abstinence at 90-day follow-up, adherence to the program, and readiness to quit."

1b-iv) RESULTS section in abstract must contain use data

Report number of participants enrolled/assessed in each group, the use/uptake of the intervention (e.g., attrition/adherence metrics, use over time, number of logins etc.), in addition to primary/secondary outcomes. (Note: Only report in the abstract what the main paper is reporting. If this information is missing from the main body of text, consider adding it)

	1	2	3	4	5	
subitem not at all important	○	○	○	○	◉	essential

Does your paper address subitem 1b-iv?

Copy and paste relevant sections from the manuscript abstract (include quotes in quotation marks "like this" to indicate direct quotes from your manuscript), or elaborate on this item by providing additional information not in the ms, or briefly explain why the item is not applicable/relevant for your study

"Follow-up rates at day-1 were 93% (56/60) for the TG and 100% (60/60) for the CG. At post-intervention, the TG reported 23.3% (14/60) abstinence on that day compared to 5.0% (3/60) in the CG. This difference was statistically significant (Chi2 (1) =8.3; P = .004). The TG reported a sustained abstinence of 33% (20/60) at 90 days. Since only 20% (12/60) participants in the CG completed the 90-day follow-up, we did not conduct a statistical comparison between groups at this follow-up time-point. Among participants still smoking at post-intervention, the TG was significantly more ready to quit compared to the CG (TG: M = 7.71; SD = 0.13; CG: M = 7.16; SD = 0.13; P = 0.005). A total of 41.1% (23/56) of participants completed the treatment in the time frame recommended by the program."

1b-v) CONCLUSIONS/DISCUSSION in abstract for negative trials

Conclusions/Discussions in abstract for negative trials: Discuss the primary outcome - if the trial is negative (primary outcome not changed), and the intervention was not used, discuss whether negative results are attributable to lack of uptake and discuss reasons. (Note: Only report in the abstract what the main paper is reporting. If this information is missing from the main body of text, consider adding it)

	1	2	3	4	5	
subitem not at all important	○	○	◉	○	○	essential

Does your paper address subitem 1b-v?

Copy and paste relevant sections from the manuscript abstract (include quotes in quotation marks "like this" to indicate direct quotes from your manuscript), or elaborate on this item by providing additional information not in the ms, or briefly explain why the item is not applicable/relevant for your study

"feasibility of trial follow-up assessment procedures for control participants needs to be improved. Further research is needed on the VR-MET influence over long-term outcomes."

INTRODUCTION

2a) In INTRODUCTION: Scientific background and explanation of rationale

2a-i) Problem and the type of system/solution

Describe the problem and the type of system/solution that is object of the study: intended as stand-alone intervention vs. incorporated in broader health care program? Intended for a particular patient population? Goals of the intervention, e.g., being more cost-effective to other interventions, replace or complement other solutions? (Note: Details about the intervention are provided in “Methods” under 5)

	1	2	3	4	5	
subitem not at all important	○	○	○	○	◉	essential

Does your paper address subitem 2a-i? *

Copy and paste relevant sections from the manuscript (include quotes in quotation marks "like this" to indicate direct quotes from your manuscript), or elaborate on this item by providing additional information not in the ms, or briefly explain why the item is not applicable/relevant for your study

"Tobacco use remains one of the biggest threats to public health and the leading preventable cause of mortality and morbidity worldwide [1]."
"Smartphone-based smoking cessation apps can provide an important channel for offering interventions to the entire population [17]. However, participant adherence remains a challenge, and many of these apps struggle to maintain high levels of adherence, thus limiting their potential effectiveness [18]."
"It has been demonstrated that the introduction of VR into treatment can improve patient engagement in a range of chronic disease interventions [21]. However, such technology has never been tested for tobacco use disorder (TUD) as a self-assisted and remote solution, taking advantage of the knowledge on how virtual environments can be used to elicit and reduce cravings and support smoking cessation."
"The aim of the current study was to develop an accessible and cost-effective digital intervention for smoking cessation that utilizes the latest technology adapted for large-scale use, and to evaluate participant adherence and smoking cessation outcomes."

2a-ii) Scientific background, rationale: What is known about the (type of) system

Scientific background, rationale: What is known about the (type of) system that is the object of the study (be sure to discuss the use of similar systems for other conditions/diagnoses, if appropiate), motivation for the study, i.e. what are the reasons for and what is the context for this specific study, from which stakeholder viewpoint is the study performed, potential impact of findings [2]. Briefly justify the choice of the comparator.

	1	2	3	4	5	
subitem not at all important	○	○	○	○	◉	essential

Does your paper address subitem 2a-ii? *

Copy and paste relevant sections from the manuscript (include quotes in quotation marks "like this" to indicate direct quotes from your manuscript), or elaborate on this item by providing additional information not in the ms, or briefly explain why the item is not applicable/relevant for your study

"In addition to smartphone-based interventions for smoking cessation, there have been promising developments in the use of virtual reality (VR). The use of VR in digital medicine has been applied in consultancy and hospitals and accompanied by a health professional [19]. It has been demonstrated that the introduction of VR into treatment can improve patient engagement in a range of chronic disease interventions [21]. However, such technology has never been tested for tobacco use disorder (TUD) as a self-assisted and remote solution, taking advantage of the knowledge on how virtual environments can be used to elicit and reduce cravings and support smoking cessation."
"Virtual Reality Exposure Therapy (VRET) has already been used for smoking cessation [32,33] and studies have applied rigorous systematizations in VRET and Cognitive-Behavior Therapy (CBT) [34–37], providing further evidence that this technique can reduce craving and smoking behavior, with similar effectiveness as standalone CBT."
"Mindfulness training is already utilized in Mobile Health (mHealth) smoking cessation interventions [39] by teaching individuals to pay attention to the present moment, understand affective states and cravings to smoke as they appear, consciously choosing to let them pass without impulsively reacting to them."

2b) In INTRODUCTION: Specific objectives or hypotheses

Does your paper address CONSORT subitem 2b? *

Copy and paste relevant sections from the manuscript (include quotes in quotation marks "like this" to indicate direct quotes from your manuscript), or elaborate on this item by providing additional information not in the ms, or briefly explain why the item is not applicable/relevant for your study

"We hypothesized that this novel intervention approach would increase both adherence and abstinence rates among participating smokers compared to a treatment as usual control group."

METHODS

3a) Description of trial design (such as parallel, factorial) including allocation ratio

Does your paper address CONSORT subitem 3a? *

Copy and paste relevant sections from the manuscript (include quotes in quotation marks "like this" to indicate direct quotes from your manuscript), or elaborate on this item by providing additional information not in the ms, or briefly explain why the item is not applicable/relevant for your study

"The design of the study follows the recommendations for clinical trials in health using virtual reality, in particular, Tier VR 2 [42], focusing on acceptability, feasibility, tolerability, and initial clinical efficacy. We conducted a clinical trial with a control group, including baseline and follow-up assessments at days 1 and 90 post-treatment."
"A total of 150 participants were accepted after establishing contact and verifying their responses. However, 30 of these individuals did not con rm their participation, which resulted in a final sample of 120 volunteers. Participants were randomized into TG (60) and CG (60) 1 : 1 using a blocked random assignment sequence."

3b) Important changes to methods after trial commencement (such as eligibility criteria), with reasons

Does your paper address CONSORT subitem 3b? *

Copy and paste relevant sections from the manuscript (include quotes in quotation marks "like this" to indicate direct quotes from your manuscript), or elaborate on this item by providing additional information not in the ms, or briefly explain why the item is not applicable/relevant for your study

No changes were made after trial commencement.

3b-i) Bug fixes, Downtimes, Content Changes

Bug fixes, Downtimes, Content Changes: ehealth systems are often dynamic systems. A description of changes to methods therefore also includes important changes made on the intervention or comparator during the trial (e.g., major bug fixes or changes in the functionality or content) (5-iii) and other "unexpected events" that may have influenced study design such as staff changes, system failures/downtimes, etc. [2].

	1	2	3	4	5	
subitem not at all important	(●)	( )	( )	( )	( )	essential

Does your paper address subitem 3b-i?

Copy and paste relevant sections from the manuscript (include quotes in quotation marks "like this" to indicate direct quotes from your manuscript), or elaborate on this item by providing additional information not in the ms, or briefly explain why the item is not applicable/relevant for your study

Item not applicable for this study. The content did not change, nor major bugs appeared during the study.

4a) Eligibility criteria for participants

Does your paper address CONSORT subitem 4a? *

Copy and paste relevant sections from the manuscript (include quotes in quotation marks "like this" to indicate direct quotes from your manuscript), or elaborate on this item by providing additional information not in the ms, or briefly explain why the item is not applicable/relevant for your study

"Age range of 24 to 65 years, a minimum consumption of 5 cigarettes per day, with a score of 4-9 on the Contemplation Scale (CL) [44], residents in the city of Buenos Aires, own an Android mobile phone with gyroscope, data plan or Wi-Fi access, and an interest in using VR as a method to quit smoking. Each of these criteria were based on previous work with mobile applications for smoking cessation [45]. The one difference in our inclusion criteria compared to this existing research was that we also included smokers that scored below 7 on the Contemplation Scale, in order to investigate intervention effects for smokers not motivated to quit."

4a-i) Computer / Internet literacy

Computer / Internet literacy is often an implicit “de facto” eligibility criterion - this should be explicitly clarified.

	1	2	3	4	5	
subitem not at all important	○	●	○	○	○	essential

Does your paper address subitem 4a-i?

Copy and paste relevant sections from the manuscript (include quotes in quotation marks "like this" to indicate direct quotes from your manuscript), or elaborate on this item by providing additional information not in the ms, or briefly explain why the item is not applicable/relevant for your study

"The treatment group was invited to the research site (University of Flores) and all participants signed an informed consent form, underwent the face-to-face on-boarding process with one member of the study team and were given the Mindcotine® Kit to begin the study."

4a-ii) Open vs. closed, web-based vs. face-to-face assessments:

Open vs. closed, web-based vs. face-to-face assessments: Mention how participants were recruited (online vs. offline), e.g., from an open access website or from a clinic, and clarify if this was a purely web-based trial, or there were face-to-face components (as part of the intervention or for assessment), i.e., to what degree got the study team to know the participant. In online-only trials, clarify if participants were quasi-anonymous and whether having multiple identities was possible or whether technical or logistical measures (e.g., cookies, email confirmation, phone calls) were used to detect/prevent these.

	1	2	3	4	5	
subitem not at all important	○	○	○	○	●	essential

Does your paper address subitem 4a-ii? *

Copy and paste relevant sections from the manuscript (include quotes in quotation marks "like this" to indicate direct quotes from your manuscript), or elaborate on this item by providing additional information not in the ms, or briefly explain why the item is not applicable/relevant for your study

"For the recruitment of treatment and control group participants, unpaid advertisements were posted for 75 days on the Facebook site of Mindcotine®, and a 13-minute segment was aired on Argentine national public television (channel C5N). The social network advertisements linked invitations through a link led directly to a screening questionnaire on the Typeform platform [46], which 1080 potentially eligible volunteers from all over Argentina completed. Of these initial applicants, 234 subjects lived in the city of Buenos Aires, and fulfilled all other necessary inclusion criteria, and telephone contact was established to verify the data. A total of 150 participants were accepted after establishing contact and verifying their responses. However, 30 of these individuals did not confirm their participation, which resulted in a final sample of 120 volunteers. Participants were randomized into TG (60) and CG (60) 1 : 1 using a blocked random assignment sequence. The treatment group was invited to the research site (University of Flores) and all participants signed an informed consent form, underwent the face-to-face on-boarding process with one member of the study team and were given the Mindcotine® Kit to begin the study."

4a-iii) Information giving during recruitment

Information given during recruitment. Specify how participants were briefed for recruitment and in the informed consent procedures (e.g., publish the informed consent documentation as appendix, see also item X26), as this information may have an effect on user self-selection, user expectation and may also bias results.

	1	2	3	4	5	
subitem not at all important	○	○	●	○	○	essential

Does your paper address subitem 4a-iii?

Copy and paste relevant sections from the manuscript (include quotes in quotation marks "like this" to indicate direct quotes from your manuscript), or elaborate on this item by providing additional information not in the ms, or briefly explain why the item is not applicable/relevant for your study

During the national TV appearance the Mindcotine intervention was explained (https://youtu.be/laV7GqC5n7U), so the people that registered from that source (more than 700 registrations in 2 hours) knew what was it about. In the Informed Consent the process is also explained, so the participant knew what to expect.

4b) Settings and locations where the data were collected

Does your paper address CONSORT subitem 4b? *

Copy and paste relevant sections from the manuscript (include quotes in quotation marks "like this" to indicate direct quotes from your manuscript), or elaborate on this item by providing additional information not in the ms, or briefly explain why the item is not applicable/relevant for your study

"Daily self-reports: At the end of the day, each user reported on the app their total daily number of cigarettes, reasons that triggered cravings, and provided a written answer to the question: "What do you think has changed in your relationship to smoking as of today?" (Nightly reflections). This data was collected on the Typeform platform embedded in the app."

4b-i) Report if outcomes were (self-)assessed through online questionnaires

Clearly report if outcomes were (self-)assessed through online questionnaires (as common in web-based trials) or otherwise.

	1	2	3	4	5	
subitem not at all important	○	○	○	○	●	essential

Does your paper address subitem 4b-i? *

Copy and paste relevant sections from the manuscript (include quotes in quotation marks "like this" to indicate direct quotes from your manuscript), or elaborate on this item by providing additional information not in the ms, or briefly explain why the item is not applicable/relevant for your study

"Daily self-reports: At the end of the day, each user reported on the app their total daily number of cigarettes, reasons that triggered cravings, and provided a written answer to the question: “What do you think has changed in your relationship to smoking as of today?” (Nightly reflections). This data was collected on the Typeform platform embedded in the app."

4b-ii) Report how institutional affiliations are displayed

Report how institutional affiliations are displayed to potential participants [on ehealth media], as affiliations with prestigious hospitals or universities may affect volunteer rates, use, and reactions with regards to an intervention.(Not a required item – describe only if this may bias results)

	1	2	3	4	5	
subitem not at all important	○	◉	○	○	○	essential

Does your paper address subitem 4b-ii?

Copy and paste relevant sections from the manuscript (include quotes in quotation marks "like this" to indicate direct quotes from your manuscript), or elaborate on this item by providing additional information not in the ms, or briefly explain why the item is not applicable/relevant for your study

During the on-boarding process, a banner was displayed showing the partnership between Mindcotine, Universidad de Flores, and School System Argentina.

5) The interventions for each group with sufficient details to allow replication, including how and when they were actually administered

5-i) Mention names, credential, affiliations of the developers, sponsors, and owners

Mention names, credential, affiliations of the developers, sponsors, and owners [6] (if authors/evaluators are owners or developer of the software, this needs to be declared in a “Conflict of interest” section or mentioned elsewhere in the manuscript).

	1	2	3	4	5	
subitem not at all important	○	○	◉	○	○	essential

Does your paper address subitem 5-i?

Copy and paste relevant sections from the manuscript (include quotes in quotation marks "like this" to indicate direct quotes from your manuscript), or elaborate on this item by providing additional information not in the ms, or briefly explain why the item is not applicable/relevant for your study

All affiliations are described in the Conflict of interest section. The tasks each member of the research had is described in the Acknowledgment section.

5-ii) Describe the history/development process

Describe the history/development process of the application and previous formative evaluations (e.g., focus groups, usability testing), as these will have an impact on adoption/use rates and help with interpreting results.

	1	2	3	4	5	
subitem not at all important	○	◉	○	○	○	essential

Does your paper address subitem 5-ii?

Copy and paste relevant sections from the manuscript (include quotes in quotation marks "like this" to indicate direct quotes from your manuscript), or elaborate on this item by providing additional information not in the ms, or briefly explain why the item is not applicable/relevant for your study

Intervention development was conducted iteratively using face-to-face testing and focus groups with feedback of 250 smokers who tested the virtual reality environment of the intervention. Moreover, we reviewed existing smoking cessation apps and features related to trigger identification and reflective questions.

5-iii) Revisions and updating

Revisions and updating. Clearly mention the date and/or version number of the application/intervention (and comparator, if applicable) evaluated, or describe whether the intervention underwent major changes during the evaluation process, or whether the development and/or content was "frozen" during the trial. Describe dynamic components such as news feeds or changing content which may have an impact on the replicability of the intervention (for unexpected events see item 3b).

	1	2	3	4	5	
subitem not at all important	(●)	( )	( )	( )	( )	essential

Does your paper address subitem 5-iii?

Copy and paste relevant sections from the manuscript (include quotes in quotation marks "like this" to indicate direct quotes from your manuscript), or elaborate on this item by providing additional information not in the ms, or briefly explain why the item is not applicable/relevant for your study

The content was the same for all participants, and there was not changes during the study.

5-iv) Quality assurance methods

Provide information on quality assurance methods to ensure accuracy and quality of information provided [1], if applicable.

	1	2	3	4	5	
subitem not at all important	( )	( )	(●)	( )	( )	essential

Does your paper address subitem 5-iv?

Copy and paste relevant sections from the manuscript (include quotes in quotation marks "like this" to indicate direct quotes from your manuscript), or elaborate on this item by providing additional information not in the ms, or briefly explain why the item is not applicable/relevant for your study

Information was collected through the platforms Amplitude and Typeform, all connected and embedded with the app.

5-v) Ensure replicability by publishing the source code, and/or providing screenshots/screen-capture video, and/or providing flowcharts of the algorithms used

Ensure replicability by publishing the source code, and/or providing screenshots/screen-capture video, and/or providing flowcharts of the algorithms used. Replicability (i.e., other researchers should in principle be able to replicate the study) is a hallmark of scientific reporting.

	1	2	3	4	5	
subitem not at all important	○	○	◉	○	○	essential

Does your paper address subitem 5-v?

Copy and paste relevant sections from the manuscript (include quotes in quotation marks "like this" to indicate direct quotes from your manuscript), or elaborate on this item by providing additional information not in the ms, or briefly explain why the item is not applicable/relevant for your study

Figures and images provide relevant information. Also it is possible to download the current version (number 3) from either Apple or Google play store.

5-vi) Digital preservation

Digital preservation: Provide the URL of the application, but as the intervention is likely to change or disappear over the course of the years; also make sure the intervention is archived (Internet Archive, webcitation.org, and/or publishing the source code or screenshots/videos alongside the article). As pages behind login screens cannot be archived, consider creating demo pages which are accessible without login.

	1	2	3	4	5	
subitem not at all important	○	◉	○	○	○	essential

Does your paper address subitem 5-vi?

Copy and paste relevant sections from the manuscript (include quotes in quotation marks "like this" to indicate direct quotes from your manuscript), or elaborate on this item by providing additional information not in the ms, or briefly explain why the item is not applicable/relevant for your study

It is possible to download the new version from Google or Apple store, with the same structure but new content. https://play.google.com/store/apps/details?id=com.mindcotineandroid&hl=en_US

5-vii) Access

Access: Describe how participants accessed the application, in what setting/context, if they had to pay (or were paid) or not, whether they had to be a member of specific group. If known, describe how participants obtained "access to the platform and Internet" [1]. To ensure access for editors/reviewers/readers, consider to provide a "backdoor" login account or demo mode for reviewers/readers to explore the application (also important for archiving purposes, see vi).

	1	2	3	4	5	
subitem not at all important	○	○	○	○	◉	essential

Does your paper address subitem 5-vii? *

Copy and paste relevant sections from the manuscript (include quotes in quotation marks "like this" to indicate direct quotes from your manuscript), or elaborate on this item by providing additional information not in the ms, or briefly explain why the item is not applicable/relevant for your study

"The intervention group completed the on-boarding process, which included digital informed consent, and received an intervention kit, including guidelines that explain the use of the program, a unique ID to access the app, a cardboard headset, three stickers to announce smoke-free areas, and two wrist-bracelets as a behavior replacement method which smokers could use to snap on their wrists when faced with a craving to smoke. Participants were also instructed to download the mobile application from the Google Play Store. The participants were trained in assembling the cardboard headset (Multimedia Appendix 1: Instructional video), use of the app, completed the baseline assessment and received an explanation on the activities to be carried out during the 21-day intervention phase."
"Participants did not receive any incentives."

5-viii) Mode of delivery, features/functionalities/components of the intervention and comparator, and the theoretical framework

Describe mode of delivery, features/functionalities/components of the intervention and comparator, and the theoretical framework [6] used to design them (instructional strategy [1], behaviour change techniques, persuasive features, etc., see e.g., [7, 8] for terminology). This includes an in-depth description of the content (including where it is coming from and who developed it) [1],” whether [and how] it is tailored to individual circumstances and allows users to track their progress and receive feedback” [6]. This also includes a description of communication delivery channels and – if computer-mediated communication is a component – whether communication was synchronous or asynchronous [6]. It also includes information on presentation strategies [1], including page design principles, average amount of text on pages, presence of hyperlinks to other resources, etc. [1].

	1	2	3	4	5	
subitem not at all important	○	○	○	○	◉	essential

Does your paper address subitem 5-viii? *

Copy and paste relevant sections from the manuscript (include quotes in quotation marks "like this" to indicate direct quotes from your manuscript), or elaborate on this item by providing additional information not in the ms, or briefly explain why the item is not applicable/relevant for your study

"The application consists of a 21-day treatment that includes 2 main activities each day, which become available after completing the activities of the previous day. (See Figure 1)
The elements of the program are:

1) Practice sessions informal mindfulness:
2a) Practice sessions in informal mindfulness using VR
2b) VR-MET design
3) Daily self-reports
4) Peer-to-peer support
5) Mindcotine® support"

5-ix) Describe use parameters

Describe use parameters (e.g., intended “doses” and optimal timing for use). Clarify what instructions or recommendations were given to the user, e.g., regarding timing, frequency, heaviness of use, if any, or was the intervention used ad libitum.

	1	2	3	4	5	
subitem not at all important	○	○	○	●	○	essential

Does your paper address subitem 5-ix?

Copy and paste relevant sections from the manuscript (include quotes in quotation marks "like this" to indicate direct quotes from your manuscript), or elaborate on this item by providing additional information not in the ms, or briefly explain why the item is not applicable/relevant for your study

"The application consists of a 21-day treatment that includes 2 main activities each day, which become available after completing the activities of the previous day."

5-x) Clarify the level of human involvement

Clarify the level of human involvement (care providers or health professionals, also technical assistance) in the e-intervention or as co-intervention (detail number and expertise of professionals involved, if any, as well as "type of assistance offered, the timing and frequency of the support, how it is initiated, and the medium by which the assistance is delivered". It may be necessary to distinguish between the level of human involvement required for the trial, and the level of human involvement required for a routine application outside of a RCT setting (discuss under item 21 – generalizability).

	1	2	3	4	5	
subitem not at all important	○	○	○	◉	○	essential

Does your paper address subitem 5-x?

Copy and paste relevant sections from the manuscript (include quotes in quotation marks "like this" to indicate direct quotes from your manuscript), or elaborate on this item by providing additional information not in the ms, or briefly explain why the item is not applicable/relevant for your study

"The treatment was remotely self-administered through the use of a mobile application."
"The treatment group was invited to the research site (University of Flores) and all participants signed an informed consent form, underwent the face-to-face on- boarding process with one member of the study team and were given the Mindcotine® Kit to begin the study."
"Peer-to-peer support: The app contained a group chat feature for interacting with all other participants. The group chat was moderated by a psychologist and a mindfulness facilitator to promote engagement and respond to participant questions."
"Mindcotine® support: If participants were inactive for a certain amount of time, they received a text message (after 2 days) and a phone call (after 4 days) to encourage engagement within the program. Participants could contact for technical support by email anytime as well."

5-xi) Report any prompts/reminders used

Report any prompts/reminders used: Clarify if there were prompts (letters, emails, phone calls, SMS) to use the application, what triggered them, frequency etc. It may be necessary to distinguish between the level of prompts/reminders required for the trial, and the level of prompts/reminders for a routine application outside of a RCT setting (discuss under item 21 – generalizability).

	1	2	3	4	5	
subitem not at all important	○	○	○	○	◉	essential

Does your paper address subitem 5-xi? *

Copy and paste relevant sections from the manuscript (include quotes in quotation marks "like this" to indicate direct quotes from your manuscript), or elaborate on this item by providing additional information not in the ms, or briefly explain why the item is not applicable/relevant for your study

"If participants were inactive for a certain amount of time, they received a text message (after 2 days) and a phone call (after 4 days) to encourage engagement within the program. Participants could contact for technical support by email anytime as well."

5-xii) Describe any co-interventions (incl. training/support)

Describe any co-interventions (incl. training/support): Clearly state any interventions that are provided in addition to the targeted eHealth intervention, as ehealth intervention may not be designed as stand-alone intervention. This includes training sessions and support [1]. It may be necessary to distinguish between the level of training required for the trial, and the level of training for a routine application outside of a RCT setting (discuss under item 21 – generalizability.

	1	2	3	4	5	
subitem not at all important	○	○	○	○	◉	essential

Does your paper address subitem 5-xii? *

Copy and paste relevant sections from the manuscript (include quotes in quotation marks "like this" to indicate direct quotes from your manuscript), or elaborate on this item by providing additional information not in the ms, or briefly explain why the item is not applicable/relevant for your study

"Mindcotine® support: If participants were inactive for a certain amount of time, they received a text message (after 2 days) and a phone call (after 4 days) to encourage engagement within the program. Participants could contact for technical support by email anytime as well."

6a) Completely defined pre-specified primary and secondary outcome measures, including how and when they were assessed

Does your paper address CONSORT subitem 6a? *

Copy and paste relevant sections from the manuscript (include quotes in quotation marks "like this" to indicate direct quotes from your manuscript), or elaborate on this item by providing additional information not in the ms, or briefly explain why the item is not applicable/relevant for your study

"Primary outcome
The primary outcome was self-reported abstinence at post-intervention, assessed 1-day after the end of the program. The question used for assessment was “Did you smoke tobacco in the last 24 hours?”

Secondary outcomes

Sustained abstinence
Sustained abstinence was self-reported abstinence at 90 days after the end of the program in the TG. The question used for assessment was “Did you smoke tobacco in the last 90 days?”

Adherence
Adherence was assessed using log file data collected employing the platform Amplitude [53], which measured total use of the app for each participant. Adherence to treatment was categorized as the percentage of participants who completed all daily activities and all daily diary questionnaires throughout the intervention; all of them with intervals of discontinuity no greater than 4 consecutive days. We operationalized full adherence as whether or not the participant completed the treatment in the suggested time (i.e., 21 days without any breaks); regular adherence as if participants completed all daily activities in up to 60 days; and depth of adherence as the total number of mindfulness training minutes [54]. Chat room activity beyond the initial on-boarding post was measured by number of participant comments in the chat room and dichotomized to any activity vs. no activity.

Cigarette consumption
Participants were instructed to report the number of cigarettes they smoked each day during treatment, as part of their daily self-reports, at post-intervention and 90-day follow-up. Over the treatment period, the daily number of cigarettes was averaged every week to generate an average number for that period.

Craving
Craving was assessed using the Questionnaire for Smoking Urges (QSU) [55], which consists of a seven-point Likert scale. This data was collected through an online survey at baseline, at the end of days 7 and 14, and at post-intervention. The internal consistency for the overall scale was adequate (α = .87).

Mindfulness
Mindfulness was assessed using the Five Facets Mindfulness Questionnaire (FFMQ) [56]. The five dimensions are: observing, describing, acting with awareness, not judging internal experience, and not reacting to internal experience. This data was collected through an online survey at baseline, at post-intervention, and at 90-day follow-up.

Readiness to quit

Readiness to quit
Readiness to quit was assessed using the Contemplation Ladder [44], which consists of 11 rungs and 5 anchor statements reflecting the stages of change, designed to measure readiness to quit smoking. It was assessed through an online survey at baseline and at post-intervention.

Nicotine Dependence
Nicotine dependence was assessed using the Fagerström Test (FTND) [57], which consists of a six-item self-report scale and observes responses suggestive of physiological dependence on nicotine. This data was collected through an online survey at the beginning of the program, at the end of day 7, 14, at post-intervention and 90-day follow-up. The internal consistency for the overall scale was adequate (α = .81)."

6a-i) Online questionnaires: describe if they were validated for online use and apply CHERRIES items to describe how the questionnaires were designed/deployed

If outcomes were obtained through online questionnaires, describe if they were validated for online use and apply CHERRIES items to describe how the questionnaires were designed/deployed [9].

	1	2	3	4	5	
subitem not at all important	(●)	( )	( )	( )	( )	essential

Does your paper address subitem 6a-i?

Copy and paste relevant sections from manuscript text

All questionnaires were collected using Typeform surveys embedded in the app.

6a-ii) Describe whether and how “use” (including intensity of use/dosage) was defined/measured/monitored

Describe whether and how “use” (including intensity of use/dosage) was defined/measured/monitored (logins, logfile analysis, etc.). Use/adoption metrics are important process outcomes that should be reported in any ehealth trial.

	1	2	3	4	5	
subitem not at all important	○	○	○	○	◉	essential

Does your paper address subitem 6a-ii?

Copy and paste relevant sections from manuscript text

"Adherence was assessed using log file data collected employing the platform Amplitude [53], which measured total use of the app for each participant. Adherence to treatment was categorized as the percentage of participants who completed all daily activities and all daily diary questionnaires throughout the intervention; all of them with intervals of discontinuity no greater than 4 consecutive days. We operationalized full adherence as whether or not the participant completed the treatment in the suggested time (i.e., 21 days without any breaks); regular adherence as if participants completed all daily activities in up to 60 days; and depth of adherence as the total number of mindfulness training minutes [54]. Chat room activity beyond the initial on-boarding post was measured by number of participant comments in the chat room and dichotomized to any activity vs. no activity."

6a-iii) Describe whether, how, and when qualitative feedback from participants was obtained

Describe whether, how, and when qualitative feedback from participants was obtained (e.g., through emails, feedback forms, interviews, focus groups).

	1	2	3	4	5	
subitem not at all important	○	◉	○	○	○	essential

Does your paper address subitem 6a-iii?

Copy and paste relevant sections from manuscript text

Qualitative feedback was collected through a face-to-face semi-structured interview at the Universidad de Flores for those participants that agreed to it once they finished the study.

6b) Any changes to trial outcomes after the trial commenced, with reasons

Does your paper address CONSORT subitem 6b? *

Copy and paste relevant sections from the manuscript (include quotes in quotation marks "like this" to indicate direct quotes from your manuscript), or elaborate on this item by providing additional information not in the ms, or briefly explain why the item is not applicable/relevant for your study

Not applicable to the study since from the beginning of it the outcomes were clearly defined.

7a) How sample size was determined

NPT: When applicable, details of whether and how the clustering by care provides or centers was addressed

7a-i) Describe whether and how expected attrition was taken into account when calculating the sample size

Describe whether and how expected attrition was taken into account when calculating the sample size.

	1	2	3	4	5	
subitem not at all important	○	◉	○	○	○	essential

Does your paper address subitem 7a-i?

Copy and paste relevant sections from manuscript title (include quotes in quotation marks "like this" to indicate direct quotes from your manuscript), or elaborate on this item by providing additional information not in the ms, or briefly explain why the item is not applicable/relevant for your study

We expected attrition to be not superior to 20%, taking into account the knowledge they had prior to starting the study on the utilization of VR and the expectation that caused them.

7b) When applicable, explanation of any interim analyses and stopping guidelines

Does your paper address CONSORT subitem 7b? *

Copy and paste relevant sections from the manuscript (include quotes in quotation marks "like this" to indicate direct quotes from your manuscript), or elaborate on this item by providing additional information not in the ms, or briefly explain why the item is not applicable/relevant for your study

There wasn't any interim analyses during the recruitment and enrollment process.

8a) Method used to generate the random allocation sequence

NPT: When applicable, how care providers were allocated to each trial group

Does your paper address CONSORT subitem 8a? *

Copy and paste relevant sections from the manuscript (include quotes in quotation marks "like this" to indicate direct quotes from your manuscript), or elaborate on this item by providing additional information not in the ms, or briefly explain why the item is not applicable/relevant for your study

"Participants were randomized into TG (60) and CG (60) 1 : 1 using a blocked random assignment sequence."

8b) Type of randomisation; details of any restriction (such as blocking and block size)

Does your paper address CONSORT subitem 8b? *

Copy and paste relevant sections from the manuscript (include quotes in quotation marks "like this" to indicate direct quotes from your manuscript), or elaborate on this item by providing additional information not in the ms, or briefly explain why the item is not applicable/relevant for your study

Block size of 2.

9) Mechanism used to implement the random allocation sequence (such as sequentially numbered containers), describing any steps taken to conceal the sequence until interventions were assigned

Does your paper address CONSORT subitem 9? *

Copy and paste relevant sections from the manuscript (include quotes in quotation marks "like this" to indicate direct quotes from your manuscript), or elaborate on this item by providing additional information not in the ms, or briefly explain why the item is not applicable/relevant for your study

"Participants were randomized into TG (60) and CG (60) 1 : 1 using a blocked random assignment sequence."

10) Who generated the random allocation sequence, who enrolled participants, and who assigned participants to interventions

Does your paper address CONSORT subitem 10? *

Copy and paste relevant sections from the manuscript (include quotes in quotation marks "like this" to indicate direct quotes from your manuscript), or elaborate on this item by providing additional information not in the ms, or briefly explain why the item is not applicable/relevant for your study

Emilio Goldenhersch generated the random allocation sequence, Cristian Waitman enrolled participants and the assignation process was randomly done when participants were verified after screening.

11a) If done, who was blinded after assignment to interventions (for example, participants, care providers, those assessing outcomes) and how

NPT: Whether or not administering co-interventions were blinded to group assignment

11a-i) Specify who was blinded, and who wasn't

Specify who was blinded, and who wasn't. Usually, in web-based trials it is not possible to blind the participants [1, 3] (this should be clearly acknowledged), but it may be possible to blind outcome assessors, those doing data analysis or those administering co-interventions (if any).

	1	2	3	4	5	
subitem not at all important	○	○	◉	○	○	essential

Does your paper address subitem 11a-i? *

Copy and paste relevant sections from the manuscript (include quotes in quotation marks "like this" to indicate direct quotes from your manuscript), or elaborate on this item by providing additional information not in the ms, or briefly explain why the item is not applicable/relevant for your study

Data analysis was done by Joaquín Ungaretti and he was blinded.

11a-ii) Discuss e.g., whether participants knew which intervention was the "intervention of interest" and which one was the "comparator"

Informed consent procedures (4a-ii) can create biases and certain expectations - discuss e.g., whether participants knew which intervention was the "intervention of interest" and which one was the "comparator".

	1	2	3	4	5	
subitem not at all important	○	◉	○	○	○	essential

Does your paper address subitem 11a-ii?

Copy and paste relevant sections from the manuscript (include quotes in quotation marks "like this" to indicate direct quotes from your manuscript), or elaborate on this item by providing additional information not in the ms, or briefly explain why the item is not applicable/relevant for your study

The intervention group knew they were receiving the intervention of interest.

11b) If relevant, description of the similarity of interventions

(this item is usually not relevant for ehealth trials as it refers to similarity of a placebo or sham intervention to a active medication/intervention)

Does your paper address CONSORT subitem 11b? *

Copy and paste relevant sections from the manuscript (include quotes in quotation marks "like this" to indicate direct quotes from your manuscript), or elaborate on this item by providing additional information not in the ms, or briefly explain why the item is not applicable/relevant for your study

Not relevant, since the intervention was a unique app utilizing virtual reality and the control was the online version of the smoking cessation handbook made and distributed by the Ministry of Health.

12a) Statistical methods used to compare groups for primary and secondary outcomes

NPT: When applicable, details of whether and how the clustering by care providers or centers was addressed

Does your paper address CONSORT subitem 12a? *

Copy and paste relevant sections from the manuscript (include quotes in quotation marks "like this" to indicate direct quotes from your manuscript), or elaborate on this item by providing additional information not in the ms, or briefly explain why the item is not applicable/relevant for your study

"Statistical analysis was performed with the Statistical Package for the Social Sciences (SPSS) for Windows (version 22, IBM, Chicago IL, USA). Abstinence rates between groups were compared using a Chi2 test. Participants with missing data at follow-up were assumed to be smoking. Repeated measure analyses of variance (ANOVAs) were used to determine between group differences and intervention effects for continuous variables. The group factor had two levels, corresponding to intervention and control group, and the Time factor had three levels, corresponding to the three assessment points (baseline, post-intervention, and 90-day follow-up). Post hoc analyses were conducted to determine significant pairwise comparisons."

12a-i) Imputation techniques to deal with attrition / missing values

Imputation techniques to deal with attrition / missing values: Not all participants will use the intervention/comparator as intended and attrition is typically high in ehealth trials. Specify how participants who did not use the application or dropped out from the trial were treated in the statistical analysis (a complete case analysis is strongly discouraged, and simple imputation techniques such as LOCF may also be problematic [4]).

	1	2	3	4	5	
subitem not at all important	○	○	◉	○	○	essential

Does your paper address subitem 12a-i? *

Copy and paste relevant sections from the manuscript (include quotes in quotation marks "like this" to indicate direct quotes from your manuscript), or elaborate on this item by providing additional information not in the ms, or briefly explain why the item is not applicable/relevant for your study

"Participants with missing data at follow-up were assumed to be smoking."

12b) Methods for additional analyses, such as subgroup analyses and adjusted analyses

Does your paper address CONSORT subitem 12b? *

Copy and paste relevant sections from the manuscript (include quotes in quotation marks "like this" to indicate direct quotes from your manuscript), or elaborate on this item by providing additional information not in the ms, or briefly explain why the item is not applicable/relevant for your study

"ANOVAs and post hoc tests were conducted to determine changes within the treatment group over time."

X26) REB/IRB Approval and Ethical Considerations [recommended as subheading under "Methods"] (not a CONSORT item)

X26-i) Comment on ethics committee approval

	1	2	3	4	5	
subitem not at all important	○	○	○	○	◉	essential

Does your paper address subitem X26-i?

Copy and paste relevant sections from the manuscript (include quotes in quotation marks "like this" to indicate direct quotes from your manuscript), or elaborate on this item by providing additional information not in the ms, or briefly explain why the item is not applicable/relevant for your study

"All study procedures were approved by the Institutional Review Board of the University of Flores, Buenos Aires, Argentina."

x26-ii) Outline informed consent procedures

Outline informed consent procedures e.g., if consent was obtained offline or online (how? Checkbox, etc.?), and what information was provided (see 4a-ii). See [6] for some items to be included in informed consent documents.

	1	2	3	4	5	
subitem not at all important	○	○	○	○	◉	essential

Does your paper address subitem X26-ii?

Copy and paste relevant sections from the manuscript (include quotes in quotation marks "like this" to indicate direct quotes from your manuscript), or elaborate on this item by providing additional information not in the ms, or briefly explain why the item is not applicable/relevant for your study

"The intervention group completed the on-boarding process, which included digital informed consent, and received an intervention kit, including guidelines that explain the use of the program, a unique ID to access the app, a cardboard headset, three stickers to announce smoke-free areas, and two wrist-bracelets as a behavior replacement method which smokers could use to snap on their wrists when faced with a craving to smoke. Participants were also instructed to download the mobile application from the Google Play Store. The participants were trained in assembling the cardboard headset (Multimedia Appendix 1: Instructional video), use of the app, completed the baseline assessment and received an explanation on the activities to be carried out during the 21-day intervention phase. The control group signed a digital informed consent sent to their emails, and received the online version of the smoking cessation manual developed by the Office of the President of the Argentine Nation [43]. Intervention group participants received the first follow-up 1 day after they completed all 21-days of intervention content, which for some participants took longer than 21 days (post-intervention follow-up) and another follow-up assessment at day 90 post-treatment. Control group participants were assessed at days 30 and 90 after they had received the smoking cessation manual. All assessment invitations were sent via email and assessments were completed online using the Typeform platform. Figure 2 shows the flow diagram of the study. Participants did not receive any "

X26-iii) Safety and security procedures

Safety and security procedures, incl. privacy considerations, and any steps taken to reduce the likelihood or detection of harm (e.g., education and training, availability of a hotline)

	1	2	3	4	5	
subitem not at all important	○	○	○	○	◉	essential

Does your paper address subitem X26-iii?

Copy and paste relevant sections from the manuscript (include quotes in quotation marks "like this" to indicate direct quotes from your manuscript), or elaborate on this item by providing additional information not in the ms, or briefly explain why the item is not applicable/relevant for your study

This information was provided in the Informed Consent.
The data will be treated with the sole purpose of improving direct attention to the user and detecting possible diagnostic and treatment methods. The data must be provided on a mandatory basis, since it is essential to identify the participant reliably, to ensure the correct process of health care. The refusal to provide the data or the inaccuracy of the data provided by the participant may cause serious inconvenience in the process of their personalized treatment. The data will be protected from uses not allowed by people outside the investigation and their confidentiality will be respected according to the Law on Protection of Personal Data, Law 25.326, sanctioned on October 4, 2000. The study will be carried out. following the ethical standards recognized by the National Mental Health Law No. 26,657, in articles 7, 10 and 16, which refer to Informed Consent.
Therefore, the information generated in this study will be considered strictly confidential, among the participating parties.

RESULTS

13a) For each group, the numbers of participants who were randomly assigned, received intended treatment, and were analysed for the primary outcome

NPT: The number of care providers or centers performing the intervention in each group and the number of patients treated by each care provider in each center

Does your paper address CONSORT subitem 13a? *

Copy and paste relevant sections from the manuscript (include quotes in quotation marks "like this" to indicate direct quotes from your manuscript), or elaborate on this item by providing additional information not in the ms, or briefly explain why the item is not applicable/relevant for your study

"At post-intervention, the TG reported 23% (14/60) abstinence on that day compared to 5% (3/60) of the CG. This difference was statistically significant (Chi2 [1] =8.3; P = .004)."

13b) For each group, losses and exclusions after randomisation, together with reasons

Does your paper address CONSORT subitem 13b? (NOTE: Preferably, this is shown in a CONSORT flow diagram) *

Copy and paste relevant sections from the manuscript (include quotes in quotation marks "like this" to indicate direct quotes from your manuscript), or elaborate on this item by providing additional information not in the ms, or briefly explain why the item is not applicable/relevant for your study

Shown in "Figure 2 Participant´s ow through Mindcotine® method smoking cessation trial".

13b-i) Attrition diagram

Strongly recommended: An attrition diagram (e.g., proportion of participants still logging in or using the intervention/comparator in each group plotted over time, similar to a survival curve) or other figures or tables demonstrating usage/dose/engagement.

	1	2	3	4	5	
subitem not at all important	○	◉	○	○	○	essential

Does your paper address subitem 13b-i?

Copy and paste relevant sections from the manuscript or cite the figure number if applicable (include quotes in quotation marks "like this" to indicate direct quotes from your manuscript), or elaborate on this item by providing additional information not in the ms, or briefly explain why the item is not applicable/relevant for your study

Not relevant to this study. The focus was mainly on adherence and initial efficacy, thus the strategies to strengthen engagement. Attrition was relatively low due to the actions taken to keep participants engaged. We are leaving usage and log details for a different article.

14a) Dates defining the periods of recruitment and follow-up

Does your paper address CONSORT subitem 14a? *

Copy and paste relevant sections from the manuscript (include quotes in quotation marks "like this" to indicate direct quotes from your manuscript), or elaborate on this item by providing additional information not in the ms, or briefly explain why the item is not applicable/relevant for your study

"The study was conducted from February to April of 2018, with a group of smokers from Buenos Aires, Argentina."
"For the recruitment of treatment and control group participants, unpaid advertisements were posted for 75 days on the Facebook site of Mindcotine®, and a 13-minute segment was aired on Argentine national public television (channel C5N)."
"Follow-up periods are 1-day after the end of the 21-days program, and 90-day after the first follow-up."

14a-i) Indicate if critical “secular events” fell into the study period

Indicate if critical “secular events” fell into the study period, e.g., significant changes in Internet resources available or “changes in computer hardware or Internet delivery resources”

	1	2	3	4	5	
subitem not at all important	(●)	( )	( )	( )	( )	essential

Does your paper address subitem 14a-i?

Copy and paste relevant sections from the manuscript (include quotes in quotation marks "like this" to indicate direct quotes from your manuscript), or elaborate on this item by providing additional information not in the ms, or briefly explain why the item is not applicable/relevant for your study

Not applicable since there weren't any secular events during the study period.

14b) Why the trial ended or was stopped (early)

Does your paper address CONSORT subitem 14b? *

Copy and paste relevant sections from the manuscript (include quotes in quotation marks "like this" to indicate direct quotes from your manuscript), or elaborate on this item by providing additional information not in the ms, or briefly explain why the item is not applicable/relevant for your study

Not applicable. The study did not end early.

15) A table showing baseline demographic and clinical characteristics for each group

NPT: When applicable, a description of care providers (case volume, qualification, expertise, etc.) and centers (volume) in each group

Does your paper address CONSORT subitem 15? *

Copy and paste relevant sections from the manuscript (include quotes in quotation marks "like this" to indicate direct quotes from your manuscript), or elaborate on this item by providing additional information not in the ms, or briefly explain why the item is not applicable/relevant for your study

"Table I. Participant baseline characteristics (N=120)"
"Participant baseline characteristics are displayed in Table I. On average, participants in the sample were 43.25 years old (SD = 10.06), and 47.5% (57/120) were female. The largest group in terms of completed educational level pertains to high school 37.5% (45/120), followed by university 35.8% (43/120). On average, the sample started smoking 19.15 years ago (SD = 12.35) and consumed an average of 10.77 (SD = 5.47) cigarettes per day. Of all participants, 14.17% (17/120) lived with other smokers in their homes. The entire sample reported a moderated nicotine dependence index based on the result obtained in the Fagerström Nicotine Dependence Test [57], score 6.83 (SD = 2.13). Only 40.8% (49/120) had practiced meditation at least once in their life. No statistically significant differences were observed between treatment and control group on any variables at baseline. "

15-i) Report demographics associated with digital divide issues

In ehealth trials it is particularly important to report demographics associated with digital divide issues, such as age, education, gender, social-economic status, computer/Internet/ehealth literacy of the participants, if known.

	1	2	3	4	5	
subitem not at all important	(●)	( )	( )	( )	( )	essential

Does your paper address subitem 15-i? *

Copy and paste relevant sections from the manuscript (include quotes in quotation marks "like this" to indicate direct quotes from your manuscript), or elaborate on this item by providing additional information not in the ms, or briefly explain why the item is not applicable/relevant for your study

It is not applicable for this study since it was known beforehand that virtual reality adoption was very low on Argentina society. This may have influenced the recruitment process from a good angle.

16) For each group, number of participants (denominator) included in each analysis and whether the analysis was by original assigned groups

16-i) Report multiple “denominators” and provide definitions

Report multiple “denominators” and provide definitions: Report N’s (and effect sizes) “across a range of study participation [and use] thresholds” [1], e.g., N exposed, N consented, N used more than x times, N used more than y weeks, N participants “used” the intervention/comparator at specific pre-defined time points of interest (in absolute and relative numbers per group). Always clearly define “use” of the intervention.

	1	2	3	4	5	
subitem not at all important	(●)	( )	( )	( )	( )	essential

Does your paper address subitem 16-i? *

Copy and paste relevant sections from the manuscript (include quotes in quotation marks "like this" to indicate direct quotes from your manuscript), or elaborate on this item by providing additional information not in the ms, or briefly explain why the item is not applicable/relevant for your study

Not applicable to this study. This information is related to a different article, where log details and usage is been leverage.

16-ii) Primary analysis should be intent-to-treat

Primary analysis should be intent-to-treat, secondary analyses could include comparing only “users”, with the appropriate caveats that this is no longer a randomized sample (see 18-i).

	1	2	3	4	5	
subitem not at all important	( )	(●)	( )	( )	( )	essential

Does your paper address subitem 16-ii?

Copy and paste relevant sections from the manuscript (include quotes in quotation marks "like this" to indicate direct quotes from your manuscript), or elaborate on this item by providing additional information not in the ms, or briefly explain why the item is not applicable/relevant for your study

"All analyses but one were intent-to-treat, analyzing “mean differences within intervention group” to comparison for users that went under the Mindcotine program.

17a) For each primary and secondary outcome, results for each group, and the estimated effect size and its precision (such as 95% confidence interval)

Does your paper address CONSORT subitem 17a? *

Copy and paste relevant sections from the manuscript (include quotes in quotation marks "like this" to indicate direct quotes from your manuscript), or elaborate on this item by providing additional information not in the ms, or briefly explain why the item is not applicable/relevant for your study

"Primary outcomes
At post-intervention, the TG reported 23% (14/60) abstinence on that day compared to 5% (3/60) of the CG. This difference was statistically significant (Chi2 [1] =8.3; P = .004).
Secondary outcomes
Sustained abstinence
The TG reported 33% (20/60) sustained abstinence on the 90-day follow-up, compared to 5% (3/60) of participants in the CG. Since only 20% (12/60) participants in the CG completed the 90-day follow-up, we did not conduct a statistical comparison between groups at this follow-up time-point.

Adherence rates
Intervention adherence was analyzed only in the TG and 93% (56/60) of participants finished the 21-day program. Of those who finished, 41.1% (23/56) were fully adherent to the program (i.e., completed all daily sessions and nightly reflections 21 days in a row) and 58.9% (33/56) were regularly adherent (completed the program in > 21 days), completing the program in 28.56 days on average. At the post-intervention, 30.4% (7/23) of fully adherent participants reported smoking abstinence, while only 21.1% (7/33) of regularly adherent participants were abstinent. At the 90-day follow-up, smoking abstinence rates were 39.1% (9/23) among fully adherent participants and 33.3% (11/33) among regularly adherent participants. No statistically significant differences were found between fully and regularly adherent groups at baseline regarding nicotine dependence (t = -.540; P = .295) and readiness to quit (t = -.483; P = .326), nor regarding abstinence rates between the two adherence groups at post-intervention (Chi2 (1) =6.1; P = .432) and at 90-day follow-up (Chi2 (1) =1.9; P = .656)
If participants had not engaged with the program for 2 subsequent days, they were contacted in order to improve engagement: 91.9% (54/60) of participants were contacted through a short message service (SMS) once; 64.9% (39/60) twice; 54.0% (32/60) thrice; and 33.9% (20/60) were contacted through both SMS and phone call reminders. Depth of adherence for all participants, measured by the number of minutes of mindfulness training during the program (including virtual reality, video format, and audio format), was 259.05 minutes on average, with a maximum of 386 and a minimum of 216 minutes.
For those who reported abstinence at post-intervention, the average of mindfulness minutes trained was 251.35 minutes, while the average was 261.62 minutes among those who continued smoking. Statistically significant differences were not found between groups (t = -1.148; P = .128).
Participant activity in the chat room beyond an initial introduction message suggested in the on-boarding process was low and only 13 participants commented in the chat room beyond the on-boarding. Chat room activity was not associated with smoking cessation outcomes, having only 1 participant that successfully quit active in the chat.

Cigarette consumption
Statistically significant differences in cigarettes per day over time were found between the TG and the CG (F (5,114) = 95.73, P < .001) in the third week of the intervention and at post [illegible]

TG and the CG (F ([illegible]) = [illegible], P < .001) in the third week of the intervention and at post-intervention. At intervention week three, the TG consumed significantly fewer cigarettes than the CG (TG: M = 6.92; SD = 5.26; CG: M = 9.03; SD = 5.42; P = .03). Significant differences in cigarettes per day between groups were also found at post-intervention (TG: M = 5.07; SD = 5.65; CG: M = 9.53; SD = 0.56; P < .001).

Readiness to quit
The means comparison in the Contemplation Scale for the TG and the CG showed statistically significant differences between groups (F (2.113) = 4.549, P = .01). Post hoc comparisons revealed that there were no differences at baseline (TG: M = 6.55; SD = 0.15; CG: M = 6.76; SD = 0.14; P = .32), but at post-intervention (TG: M = 7.71; SD = 0.13; CG: M = 7.16; SD = 0.13; P = .005)."

17a-i) Presentation of process outcomes such as metrics of use and intensity of use

In addition to primary/secondary (clinical) outcomes, the presentation of process outcomes such as metrics of use and intensity of use (dose, exposure) and their operational definitions is critical. This does not only refer to metrics of attrition (13-b) (often a binary variable), but also to more continuous exposure metrics such as “average session length”. These must be accompanied by a technical description how a metric like a “session” is defined (e.g., timeout after idle time) [1] (report under item 6a).

	1	2	3	4	5	
subitem not at all important	○	○	○	○	◉	essential

Does your paper address subitem 17a-i?

Copy and paste relevant sections from the manuscript (include quotes in quotation marks "like this" to indicate direct quotes from your manuscript), or elaborate on this item by providing additional information not in the ms, or briefly explain why the item is not applicable/relevant for your study

"Adherence rates
Intervention adherence was analyzed only in the TG and 93% (56/60) of participants finished the 21-day program. Of those who finished, 41.1% (23/56) were fully adherent to the program (i.e., completed all daily sessions and nightly reflections 21 days in a row) and 58.9% (33/56) were regularly adherent (completed the program in > 21 days), completing the program in 28.56 days on average. At the post-intervention, 30.4% (7/23) of fully adherent participants reported smoking abstinence, while only 21.1% (7/33) of regularly adherent participants were abstinent. At the 90-day follow-up, smoking abstinence rates were 39.1% (9/23) among fully adherent participants and 33.3% (11/33) among regularly adherent participants. No statistically significant differences were found between fully and regularly adherent groups at baseline regarding nicotine dependence (t = -.540; P = .295) and readiness to quit (t = -.483; P = .326), nor regarding abstinence rates between the two adherence groups at post-intervention (Chi2 (1) =6.1; P = .432) and at 90-day follow-up (Chi2 (1) =1.9; P = .656)
If participants had not engaged with the program for 2 subsequent days, they were contacted in order to improve engagement: 91.9% (54/60) of participants were contacted through a short message service (SMS) once; 64.9% (39/60) twice; 54.0% (32/60) thrice; and 33.9% (20/60) were contacted through both SMS and phone call reminders. Depth of adherence for all participants, measured by the number of minutes of mindfulness training during the program (including virtual reality, video format, and audio format), was 259.05 minutes on average, with a maximum of 386 and a minimum of 216 minutes.
For those who reported abstinence at post-intervention, the average of mindfulness minutes trained was 251.35 minutes, while the average was 261.62 minutes among those who continued smoking. Statistically significant differences were not found between groups (t = -1.148; P = .128).
Participant activity in the chat room beyond an initial introduction messages suggested in the on-boarding process was low and only 13 participants commented in the chat room beyond the on-boarding. Chat room activity was not associated with smoking cessation outcomes, having only 1 participant that successfully quit active in the chat."

17b) For binary outcomes, presentation of both absolute and relative effect sizes is recommended

Does your paper address CONSORT subitem 17b? *

Copy and paste relevant sections from the manuscript (include quotes in quotation marks "like this" to indicate direct quotes from your manuscript), or elaborate on this item by providing additional information not in the ms, or briefly explain why the item is not applicable/relevant for your study

"Primary outcomes
At post-intervention, the TG reported 23% (14/60) abstinence on that day compared to 5% (3/60) of the CG. This difference was statistically significant (Chi2 [1] =8.3; P = .004).
Secondary outcomes
Sustained abstinence
The TG reported 33% (20/60) sustained abstinence on the 90-day follow-up, compared to 5% (3/60) of participants in the CG. Since only 20% (12/60) participants in the CG completed the 90-day follow-up, we did not conduct a statistical comparison between groups at this follow-up time-point."

18) Results of any other analyses performed, including subgroup analyses and adjusted analyses, distinguishing pre-specified from exploratory

Does your paper address CONSORT subitem 18? *

Copy and paste relevant sections from the manuscript (include quotes in quotation marks "like this" to indicate direct quotes from your manuscript), or elaborate on this item by providing additional information not in the ms, or briefly explain why the item is not applicable/relevant for your study

"Mean differences within the treatment group over time
Cigarrete consumption
Post-hoc tests after ANOVA were conducted for cigarette consumption, and statistically significant differences over time were found within the TG (F (4. 52) = 13.79; P < .001). Participants significantly reduced their cigarettes per day from baseline (M = 11.09; SD = 5.27) to post-intervention (M = 6.05; SD = 5.67; P < .001), as well as from baseline to 90-day follow-up (M = 5.07; SD = 5.65; P < .001).

Mindfulness
Moreover, according to paired T test comparisons for FFMQ scores no statistically significant difference was found between baseline (M = 123.95; SD = 9.55) and post-intervention (M = 122.61; SD = 6.31; t (55) = 0.772; P = .4431). However, FFMQ at 90-day follow-up (M = 115.89; SD = 12.30) was significantly lower than scores at baseline (t (55) = 5.594; P < .001) and post-intervention (t (55) = 3.234; P = .002).

Craving
A significant reduction in self-reported craving over time was observed in the treatment group (Baseline M = 30.28, SD = 11.72; Intervention Week 1 M = 30.01, SD = 11.75; Intervention Week 2 M = 28.00, SD = 13; Post-Intervention M = 26.00, SD = 11.37). These differences over time were statistically significant (F (3.725), P = .005)."

18-i) Subgroup analysis of comparing only users

A subgroup analysis of comparing only users is not uncommon in ehealth trials, but if done, it must be stressed that this is a self-selected sample and no longer an unbiased sample from a randomized trial (see 16-iii).

	1	2	3	4	5	
subitem not at all important	○	●	○	○	○	essential

Does your paper address subitem 18-i?

Copy and paste relevant sections from the manuscript (include quotes in quotation marks "like this" to indicate direct quotes from your manuscript), or elaborate on this item by providing additional information not in the ms, or briefly explain why the item is not applicable/relevant for your study

The subgroup corresponds to the treatment group.

19) All important harms or unintended effects in each group

(for specific guidance see CONSORT for harms)

Does your paper address CONSORT subitem 19? *

Copy and paste relevant sections from the manuscript (include quotes in quotation marks "like this" to indicate direct quotes from your manuscript), or elaborate on this item by providing additional information not in the ms, or briefly explain why the item is not applicable/relevant for your study

During the face-to-face on-boarding process it was explained to the participants that the use of virtual reality may cause cyber-sickness and, even though it was part of the screening process, it was mentioned that if they suffer from epilepsy it shouldn't be used.

19-i) Include privacy breaches, technical problems

Include privacy breaches, technical problems. This does not only include physical “harm” to participants, but also incidents such as perceived or real privacy breaches [1], technical problems, and other unexpected/unintended incidents. “Unintended effects” also includes unintended positive effects [2].

	1	2	3	4	5	
subitem not at all important	(●)	○	○	○	○	essential

Does your paper address subitem 19-i?

Copy and paste relevant sections from the manuscript (include quotes in quotation marks "like this" to indicate direct quotes from your manuscript), or elaborate on this item by providing additional information not in the ms, or briefly explain why the item is not applicable/relevant for your study

Not applicable to this study. There weren't any incidents of privacy breaches.

19-ii) Include qualitative feedback from participants or observations from staff/researchers

Include qualitative feedback from participants or observations from staff/researchers, if available, on strengths and shortcomings of the application, especially if they point to unintended/unexpected effects or uses. This includes (if available) reasons for why people did or did not use the application as intended by the developers.

	1	2	3	4	5	
subitem not at all important	○	●	○	○	○	essential

Does your paper address subitem 19-ii?

Copy and paste relevant sections from the manuscript (include quotes in quotation marks "like this" to indicate direct quotes from your manuscript), or elaborate on this item by providing additional information not in the ms, or briefly explain why the item is not applicable/relevant for your study

Qualitative feedback was collected from research and focus groups and the result is the current online version of the app.
Feedback from users:
“The cardboard headset was not comfortable enough to have it 10 minutes on.” “The need to be connected to Wi to download the contents was not reliable since the signal wasn’t always good enough and that caused stress. “
“After going through some sessions I started to feel my body, my legs, my arms, and I knew I was breathing.
It is a non-invasive intervention and after trying so many things, I can't see why not to try this.”

DISCUSSION

22) Interpretation consistent with results, balancing benefits and harms, and considering other relevant evidence

NPT: In addition, take into account the choice of the comparator, lack of or partial blinding, and unequal expertise of care providers or centers in each group

22-i) Restate study questions and summarize the answers suggested by the data, starting with primary outcomes and process outcomes (use)

Restate study questions and summarize the answers suggested by the data, starting with primary outcomes and process outcomes (use).

	1	2	3	4	5	
subitem not at all important	○	○	○	○	◉	essential

Does your paper address subitem 22-i? *

Copy and paste relevant sections from the manuscript (include quotes in quotation marks "like this" to indicate direct quotes from your manuscript), or elaborate on this item by providing additional information not in the ms, or briefly explain why the item is not applicable/relevant for your study

"This is the first study to report results of a pilot trial testing a VR smartphone-based smoking cessation program using remote and self-assisted delivery. At post-intervention, the intervention group had a significantly higher abstinence rate (23% [14/60] abstinence), compared to a control group that received a smoking cessation manual (5% [3/60] abstinence). Moreover, the intervention resulted in a 33% (20/60) abstinence rate at 90-day follow-up and high levels of adherence and engagement. Findings suggest the potential efficacy of a smartphone-based VR intervention that combines exposure therapy and mindfulness for smoking cessation.
When compared to other smoking cessation app studies, abstinence rates observed in the current study were in a similar range. For instance, an 8-week single-arm trial of a smartphone app, which delivered essential features of US clinical practice guidelines with personalization, resulted in a 26% abstinence rate at 30-day follow-up [58]. Another single-arm trial based on acceptance and commitment therapy had abstinence rates of 33% at 7-day follow-up and 28% at 30-day follow-up [59]. These rates are similar to the 23% and 33% observed in our study and provide confidence for further development and testing.
With 93% of participants finishing the 21-day program, the current study had a high completion and low dropout rate. In line with other studies, we used several strategies to enhance adherence, including SMS and phone call reminders [54]. Consistent with our findings, adherence rates were also above 80% in the above-mentioned 8-week single-arm clinical trial [58] and in an RCT of a text messaging program [60]. Of all 56 TG participants who finished the program, 23 completed without any gaps in treatment adherence and were classified as fully adherent, while the other 33 participants took an average of 28.56 days to complete the program and were classified as regularly adherent. Abstinence at post-intervention and 90-day follow-up was higher among fully adherent participants compared to regularly adherent participants. These findings suggest that repeated exposure to smoking-related cues in virtual environments alongside mindfulness practice on a consecutive daily basis may increase abstinence outcomes compared to sessions that are more spaced out over time. Thus, a more consistent and disciplined training using virtual reality mindfulness-based exposure therapy to both internal and external triggers over a shorter period of time may result in better outcomes. Based on other studies [61], the Chat room activity was low probably due to the lack of promotion to increase engagement through tailored material by the moderators. Overall, the methods used to strengthen the engagement have shown to have value and can be further developed as well.
Our results also indicate that mindfulness scores measured by the FFMQ significantly decreased within the TG from baseline to 90-day follow-up. It is unclear what may have caused this decrease despite the promising smoking cessation outcomes of our intervention. Given that the current study is the first to combine virtual reality exposure therapy and mindfulness-based relapse prevention for smoking cessation, additional replication of these findings is needed. Moreover, future studies should further explore mechanisms of action of this novel intervention approach.
Other studies using VR and exposure therapy in smoking cessation showed similar results decreasing cue-induced craving, such as a randomized clinical trial that combined CBT and [illegible]

VR-CET found a significant reduction of cue-induced cravings after group-based sessions over the course of 6 months with 5 individual sessions of VR-CET [37]. In the current study, the virtual reality environments exposed participants to smoking-related cues and at the same time presented a mindfulness narrative based on relapse prevention. Therefore, the intervention simultaneously elicits cravings by means of VRET and provides the user with tools for reducing these cravings by means of mindfulness. Our results of QSU craving scores over time show that scores did not decrease during the first week of treatment, in which there was no exposure therapy. However, self-reported cravings decreased during the following 14 days of the program in which VR-MET was delivered.

Feasibility of conducting the trial was demonstrated by the fact that we were able to recruit 120 participants in the current pilot trial, however, feasibility of collecting follow-up data from a control group needs to be improved. The delivery of the intervention was feasible and acceptable to participants: 41% of intervention participants completed the entire intervention content in 21 days, as recommended, and the average adherence to mindfulness training was 259 minutes per participant. Feasibility of the VR component as a central element of the program was high, with all participants practicing at least 15 informal mindfulness practice sessions lasting 10 minutes each. Even though this intervention introduced VR and mindfulness training to a population that had almost never tried any of these approaches before, the intervention had high engagement rates, suggesting remote and self-administered VR can be used as a strategy for improving adherence to mHealth interventions. These findings are in line with those of existing studies, which have shown that VR can enhance treatment fidelity by having behavioral interventions delivered by a programmed avatar [21]; and that VR environments offer existing opportunities to enhance a patient´s involvement in treatments [62].

Given that VR in mental health to date has predominantly been used in in-patient hospital environments [20], the recruitment method used in the current study to test the potential of VR as a self-administered and remote smoking cessation intervention on an out-patient population is worth mentioning. After appearing on national television, more than 1,000 volunteers from all over Argentina registered within a few hours. The average age of these volunteers was 41 years, which was considerably older than we had anticipated. The fact that we were able to recruit this population for a smartphone-based smoking cessation study using a VR cardboard headset demonstrates that this intervention can be accessible to populations that do not need to be exceptionally tech-savvy. In light of existing challenges to recruit participants for mHealth interventions [63], this particular recruitment strategy may prove valuable for future research.

The creation of Mindcotine® involved a group of psychologists, psychiatrists and other physicians, as well as developers, actors, and mindfulness facilitators, and took over 6 months. While initial development costs were high, the fact that this program can be delivered remotely and self-administered makes it a low-cost and accessible intervention to promote smoking cessation."

22-ii) Highlight unanswered new questions, suggest future research

Highlight unanswered new questions, suggest future research.

	1	2	3	4	5	
subitem not at all important	○	○	○	◉	○	essential

Does your paper address subitem 22-ii?

Copy and paste relevant sections from the manuscript (include quotes in quotation marks "like this" to indicate direct quotes from your manuscript), or elaborate on this item by providing additional information not in the ms, or briefly explain why the item is not applicable/relevant for your study

"Our results also indicate that mindfulness scores measured by the FFMQ significantly decreased within the TG from baseline to 90-day follow-up. It is unclear what may have caused this decrease despite the promising smoking cessation outcomes of our intervention. Given that the current study is the first to combine virtual reality exposure therapy and mindfulness-based relapse prevention for smoking cessation, additional replication of these findings is needed. Moreover, future studies should further explore mechanisms of action of this novel intervention approach."
Limitations section expands on more topics for future research.

20) Trial limitations, addressing sources of potential bias, imprecision, and, if relevant, multiplicity of analyses

20-i) Typical limitations in ehealth trials

Typical limitations in ehealth trials: Participants in ehealth trials are rarely blinded. Ehealth trials often look at a multiplicity of outcomes, increasing risk for a Type I error. Discuss biases due to non-use of the intervention/usability issues, biases through informed consent procedures, unexpected events.

	1	2	3	4	5	
subitem not at all important	○	○	○	○	◉	essential

Does your paper address subitem 20-i? *

Copy and paste relevant sections from the manuscript (include quotes in quotation marks "like this" to indicate direct quotes from your manuscript), or elaborate on this item by providing additional information not in the ms, or briefly explain why the item is not applicable/relevant for your study

"The current study has several limitations. The follow-up time was relatively short, given that only between 3% and 5% of smokers remain abstinent within the first year of quitting [64]. Follow-up assessment time-point were not identical across intervention and control groups. Before the current pilot trial, we did not know how long participants would take to complete the entire program, thus we selected a 30-day follow-up time point for the control group. Future research will adhere to a consistent follow-up assessment time point across intervention and control groups. Completion of the 90-day follow-up in the control group was low. It is possible that control group participants may not have been motivated to complete follow-up surveys due to not seeing changes in their behavior, not receiving a digital intervention, or not receiving any monetary incentive. Control group follow-up rates need to be improved in future trials of this intervention. Other limitations include that at baseline, participants were not regular users of VR; the virtual intervention content was not interactive; only an Android version of the intervention app was available, and the version of the intervention tested in the current study did not include features for the users to track cravings on the app and in virtual reality environments. These features are currently in development for future versions of the intervention. Moreover, abstinence at follow-up was self-reported and subsequent investigations should include biochemical verification of outcomes.
Finally, the face-to-face meeting and the on-boarding process prior at the beginning of the program could have impacted the high adherence rates observed in this trial. Since the current study was designed as a pilot-RCT, face-to-face on-boarding was conducted to determine the most important information to be delivered in remote on-boarding in future interventions. Future studies should administer the entire program remotely."

21) Generalisability (external validity, applicability) of the trial findings

NPT: External validity of the trial findings according to the intervention, comparators, patients, and care providers or centers involved in the trial

21-i) Generalizability to other populations

Generalizability to other populations: In particular, discuss generalizability to a general Internet population, outside of a RCT setting, and general patient population, including applicability of the study results for other organizations

	1	2	3	4	5	
subitem not at all important	○	◉	○	○	○	essential

Does your paper address subitem 21-i?

Copy and paste relevant sections from the manuscript (include quotes in quotation marks "like this" to indicate direct quotes from your manuscript), or elaborate on this item by providing additional information not in the ms, or briefly explain why the item is not applicable/relevant for your study

The intervention is currently online and available for purchase. It is already been used for the general population.

21-ii) Discuss if there were elements in the RCT that would be different in a routine application setting

Discuss if there were elements in the RCT that would be different in a routine application setting (e.g., prompts/reminders, more human involvement, training sessions or other co-interventions) and what impact the omission of these elements could have on use, adoption, or outcomes if the intervention is applied outside of a RCT setting.

	1	2	3	4	5	
subitem not at all important	◉	○	○	○	○	essential

Does your paper address subitem 21-ii?

Copy and paste relevant sections from the manuscript (include quotes in quotation marks "like this" to indicate direct quotes from your manuscript), or elaborate on this item by providing additional information not in the ms, or briefly explain why the item is not applicable/relevant for your study

The paper does not address this item, but it was very relevant to continue the development of the program. The most important change is the adaptation of the UX/UI for the general population. The number of questions asked to participants during the RCT is not acceptable in an app outside of a trial setting. Regarding a number of training sessions, 2 VR contents represent a short number of environments and triggers, thus the necessity to add a more varied number of them. Both of these changes can have a clear impact on the perception of the general population when adopting a VR smartphone-based intervention for smoking cessation.

OTHER INFORMATION

23) Registration number and name of trial registry

Does your paper address CONSORT subitem 23? *

Copy and paste relevant sections from the manuscript (include quotes in quotation marks "like this" to indicate direct quotes from your manuscript), or elaborate on this item by providing additional information not in the ms, or briefly explain why the item is not applicable/relevant for your study

"Trial Registration Number: isrctn.com ID ISRCTN50586181, http://www.isrctn.com/ISRCTN50586181"

24) Where the full trial protocol can be accessed, if available

Does your paper address CONSORT subitem 24? *

Cite a Multimedia Appendix, other reference, or copy and paste relevant sections from the manuscript (include quotes in quotation marks "like this" to indicate direct quotes from your manuscript), or elaborate on this item by providing additional information not in the ms, or briefly explain why the item is not applicable/relevant for your study

Not applicable to be written in the manuscript. The full trial protocol is available by request.

25) Sources of funding and other support (such as supply of drugs), role of funders

Does your paper address CONSORT subitem 25? *

Copy and paste relevant sections from the manuscript (include quotes in quotation marks "like this" to indicate direct quotes from your manuscript), or elaborate on this item by providing additional information not in the ms, or briefly explain why the item is not applicable/relevant for your study

"This research was supported financially by the University of Flores (internal protocol number 17EX04), by a grant received from the Argentine Ministry of Production, Seed Fund project number 3291022226, and sponsored by Mindcotine® Inc. Emilio Goldenhersch and Marcelo Ceberio participated in the study design. Nicolas Rosencovich and Cristian Waitman participated in the collection process. Joaquin Ungaretti participated in the data analysis. MC, EG and Johannes Thrul participated in the interpretation of data. JT and EG participated in the writing of the paper and the decision to submit for publication. "

X27) Conflicts of Interest (not a CONSORT item)

X27-i) State the relation of the study team towards the system being evaluated

In addition to the usual declaration of interests (financial or otherwise), also state the relation of the study team towards the system being evaluated, i.e., state if the authors/evaluators are distinct from or identical with the developers/sponsors of the intervention.

	1	2	3	4	5	
subitem not at all important	○	○	○	○	●	essential

Does your paper address subitem X27-i?

Copy and paste relevant sections from the manuscript (include quotes in quotation marks "like this" to indicate direct quotes from your manuscript), or elaborate on this item by providing additional information not in the ms, or briefly explain why the item is not applicable/relevant for your study

"Joaquin Ungaretti has no conflict of interest.
Dr. Thrul's contribution to this publication was as a member of the Advisory Board of MindCotine, Inc. This arrangement has been reviewed and approved by the Johns Hopkins University in accordance with its conflict of interest policies.
Dr. Ceberio has no conflicts of interest.
All other authors have shares in Mindcotine® Inc., the company that developed the VR environments and the app for this study."

About the CONSORT EHEALTH checklist

As a result of using this checklist, did you make changes in your manuscript? *

- ○ yes, major changes
- ◉ yes, minor changes
- ○ no

What were the most important changes you made as a result of using this checklist?

The most important changes were made in the Methods sections, specifically in the Participants one.

How much time did you spend on going through the checklist INCLUDING making changes in your manuscript *

4 week-days.

As a result of using this checklist, do you think your manuscript has improved? *

- (•) yes
- ( ) no
- ( ) Other:

Would you like to become involved in the CONSORT EHEALTH group?

This would involve for example becoming involved in participating in a workshop and writing an "Explanation and Elaboration" document

- ( ) yes
- (•) no
- ( ) Other:

Any other comments or questions on CONSORT EHEALTH

Very helpful methodology to go over every little detail. I appreciate the structure and the deepness of it. One recommendation. Please ADD the number of characters or words available for each answer, since it takes a long time to fill every response and at the end, when pressing "Submit" there is an error if the answers were too long.

STOP - Save this form as PDF before you click submit

To generate a record that you filled in this form, we recommend to generate a PDF of this page (on a Mac, simply select "print" and then select "print as PDF") before you submit it.

When you submit your (revised) paper to JMIR, please upload the PDF as supplementary file.

Don't worry if some text in the textboxes is cut off, as we still have the complete information in our database. Thank you!

Final step: Click submit !

Click submit so we have your answers in our database!

Submit

Never submit passwords through Google Forms.

This content is neither created nor endorsed by Google. Report Abuse - Terms of Service - Privacy Policy

Google Forms
